# Root anatomical traits contribute to deeper rooting of maize under compacted field conditions

**DOI:** 10.1093/jxb/eraa165

**Published:** 2020-05-18

**Authors:** Dorien J Vanhees, Kenneth W Loades, A Glyn Bengough, Sacha J Mooney, Jonathan P Lynch

**Affiliations:** 1 Division of Agricultural and Environment Sciences, School of Biosciences, University of Nottingham, Sutton Bonington Campus, Leicestershire, UK; 2 The James Hutton Institute, Invergowrie, UK; 3 School of Science and Engineering, The University of Dundee, Dundee, UK; 4 Forschungszentrum Jülich, Germany

**Keywords:** Aerenchyma, cortical cell file number, compaction, rooting depth, root class, thickening

## Abstract

To better understand the role of root anatomy in regulating plant adaptation to soil mechanical impedance, 12 maize lines were evaluated in two soils with and without compaction treatments under field conditions. Penetrometer resistance was 1–2 MPa greater in the surface 30 cm of the compacted plots at a water content of 17–20% (v/v). Root thickening in response to compaction varied among genotypes and was negatively associated with rooting depth at one field site under non-compacted plots. Thickening was not associated with rooting depth on compacted plots. Genotypic variation in root anatomy was related to rooting depth. Deeper-rooting plants were associated with reduced cortical cell file number in combination with greater mid cortical cell area for node 3 roots. For node 4, roots with increased aerenchyma were deeper roots. A greater influence of anatomy on rooting depth was observed for the thinner root classes. We found no evidence that root thickening is related to deeper rooting in compacted soil; however, anatomical traits are important, especially for thinner root classes.

## Teaser

Maize root anatomy significantly contributes to deeper rooting when grown in compacted field conditions due to root class-dependent traits such as reduced cell file number and increased aerenchyma formation.

## Introduction

Mechanical impedance has important effects on root development and plant growth as it restricts soil exploration and therefore nutrient and water capture (Yamaguchi and Tanaka, 1990; Merotto and Mundstock, 1999; Lipiec and Hatano, 2003; Batey, 2009). Improved understanding of root adaptations to mechanical impedance could contribute to the development of crops with improved exploration of hard soils, commonly encountered in deep soil horizons, with improved water and nutrient acquisition (Lynch and Wojciechowski, 2015).

Root diameter often increases in response to mechanical impedance (Atwell, 1993; Pritchard, 1994; Iijima *et al.*, 2000; Tracy *et al.*, 2012; Pfeifer *et al.*, 2014; Colombi and Walter, 2016) as cell division decreases (Clark *et al.*, 2003) and root elongation slows (Atwell, 1993; Gregory, 2006). Mechanical impedance >2 MPa reduces root elongation for most plants (Atwell, 1993). The energy cost of root elongation increases with increasing penetration resistance (Colombi *et al.*, 2019). Radial thickening is thought to relieve stress from the root tip while deforming the soil near the root tip, allowing the root to penetrate deeper into the compacted soil (Hettiaratchi, 1990; Atwell, 1993; Pritchard, 1994; Kirby and Bengough, 2002; Bengough *et al.*, 2006; Gregory, 2006). Furthermore, thicker roots have been linked to increased buckling resistance (Clark *et al.*, 2003; Chimungu *et al.*, 2015*a*). Radial thickening occurs within the elongation zone immediately basal to the root tip. The elongation zone itself becomes shorter when under mechanical impedance (Croser *et al.*, 1999; Bengough *et al.*, 2006) which can reduce the friction upon this zone as the zone has become smaller (Atwell, 1993). Theoretical simulations of roots growing through a strong sandy loam soil showed that larger roots were associated with smaller shear stresses over the root surface and lower axial stress at the root tip, and that thickening as such could be of advantage to roots that experience impedance (Kirby and Bengough, 2002). Thicker roots might be beneficial, while thickening itself would only contribute to reduce stress on a localized scale at the root tip. The difference between a thicker root and a thickening root should be noted as thickening is also associated with reduced elongation rates due to anatomical changes. Mechanical impedance will induce shorter and fatter cells to be formed (Bengough *et al.*, 2006), which contribute to reduced elongation. Mechanical impedance also causes slower cell flux out of the meristem (Croser *et al.*, 1999). Reduced root elongation reduces soil exploration, while those roots that do not thicken and are able to elongate normally would be capable of acquiring more water and nutrients. Reports regarding the thickening response of specific root classes are scarce as most studies consider seedling roots. Root diameter and cross-sectional area of 2-day-old wheat seed-borne roots increased under increased soil strength up to a maximum diameter of 0.78 mm, whereas diameters of nodal roots (first node) increased less while still reaching a similar maximum (Colombi *et al.*, 2017). In another recent study (Colombi and Walter, 2016), root diameter under compaction increased in young soybean adventitious roots; however, as the plants aged, root diameter was similar between compacted and uncompacted conditions. The adaptive utility of root thickening, as opposed to the possibility that it represents reduced cell formation and elongation, remains unclear.

Root anatomical phenotypes can improve adaptation to abiotic stresses including suboptimal nitrogen (Lynch, 2013; Saengwilai *et al.*, 2014; Schneider *et al.*, 2017), phosphorus (Schneider *et al.*, 2017; Galindo-Castañeda *et al.*, 2018; Schneider and Lynch, 2018; Strock *et al.*, 2018), water deficit (Jaramillo *et al.*, 2013; Lynch, 2013, 2018; Chimungu *et al.*, 2014), as well as flooding (hypoxia; Yamauchi, 2013). Root anatomy correlated with penetration of strong wax layers in maize (Chimungu *et al.*, 2015). Thickening under impedance has been related to the changes in the underlying tissues and cellular structures. Both the cortex and the stele react to mechanical impedance. Cortical changes such as the addition of cell layers (Wilson *et al.*, 1977; Colombi *et al.* 2019) or the expansion of cortical cells have been observed (Atwell, 1988; Hanbury and Atwell, 2005; Colombi *et al*., 2017, 2019). For instance, in lupin, which is usually able to penetrate strong soil (Materechera *et al.*, 1991), radial thickening is caused by the swelling of cortical cells, rather than the addition of cell files (Atwell, 1988; Hanbury and Atwell, 2005). However, diverse observations have been made for different species and under a range of experimental conditions. A good overview of different cortical changes associated with impedance, as well as changes to vascular tissues and meristems, has been discussed recently by Potocka and Szymanowska-Pułka (2018). Additionally, increased aerenchyma area has been observed under impeded conditions (Colombi and Walter, 2016; Colombi *et al.*, 2017), which may have adaptive value (Lynch and Wojciechowski, 2015). Recent developments such as laser ablation tomography (LAT) accompanied by image analysis have enabled more rapid anatomical phenotyping (Hall *et al.*, 2019; Strock *et al.*, 2019), facilitating the analysis of multiple root classes, genotypes, and environments, to discern relationships between root anatomical phenotypes and responses to mechanical impedance.

Comparisons among species suggest that plants with thicker seedling roots are better able to penetrate hard soils (Materechera *et al.*, 1991). For pea, a 3-fold increase in maximum growth force was associated with primary versus lateral roots, with primary roots exerting 6–32% more pressure (Misra, 1997). A study of 14 wheat genotypes revealed that traits such as root cross-sectional area (RCSA), stele area, cortical area, root cortical aerenchyma, cell size, and cell file number (CF) were affected by increased soil strength, and that responses were genotype dependent as well as differing between primary and first node roots (Colombi *et al.*, 2017). How different axial root classes of maize adapt to impedance under field conditions has so far not been considered. A maize root system consists of nodal root axes characterized by increasing diameters and associated changed anatomy per node that potentially could react differently to mechanical impedance. Diameter changes associated with underlying changes in anatomy have so far not been studied across different genotypes. Different soil types, in this instance soil texture, can influence the root diameter of tomato plants as shown by Tracy *et al.* (2012). Field studies carried out on different soil types have identified differences in root distributions under impeded conditions, but anatomical differences between fields have so far not been accounted for.

Here, we propose that thickening *per se* does not explain differential rooting capabilities among maize genotypes. We hypothesize that radial thickening in response to mechanical impedance will vary among genotypes, root classes, and soil types. We propose that older, thinner nodes will thicken more than younger, thicker nodal roots. Further we hypothesize that node-specific root anatomical phenotypes influence growth through compacted soils, especially in thinner roots from older nodes, as opposed to thicker roots from younger nodes. Roots of younger nodes, that are thicker from the start, do not have a need for extensive thickening and hence fewer cellular adjustments would be needed. However, younger, thicker roots can benefit from tissue adjustments, such as the formation of aerenchyma. This can further aid root growth in compacted soils as these soils are often not just a source of impedance but are also associated with a lower level of oxygen (Lynch and Wojciechowski, 2015).

The purpose of this study was to identify whether radial thickening is related to root penetration of hard soils or rather is an indication of non-penetration, in contrasting soils, genotypes, and root classes. Secondly, we tested the hypothesis that root anatomical phenotypes contribute to growth in hard soil.

## Materials and methods

### Growth conditions and plant material

**Fig. 1.**
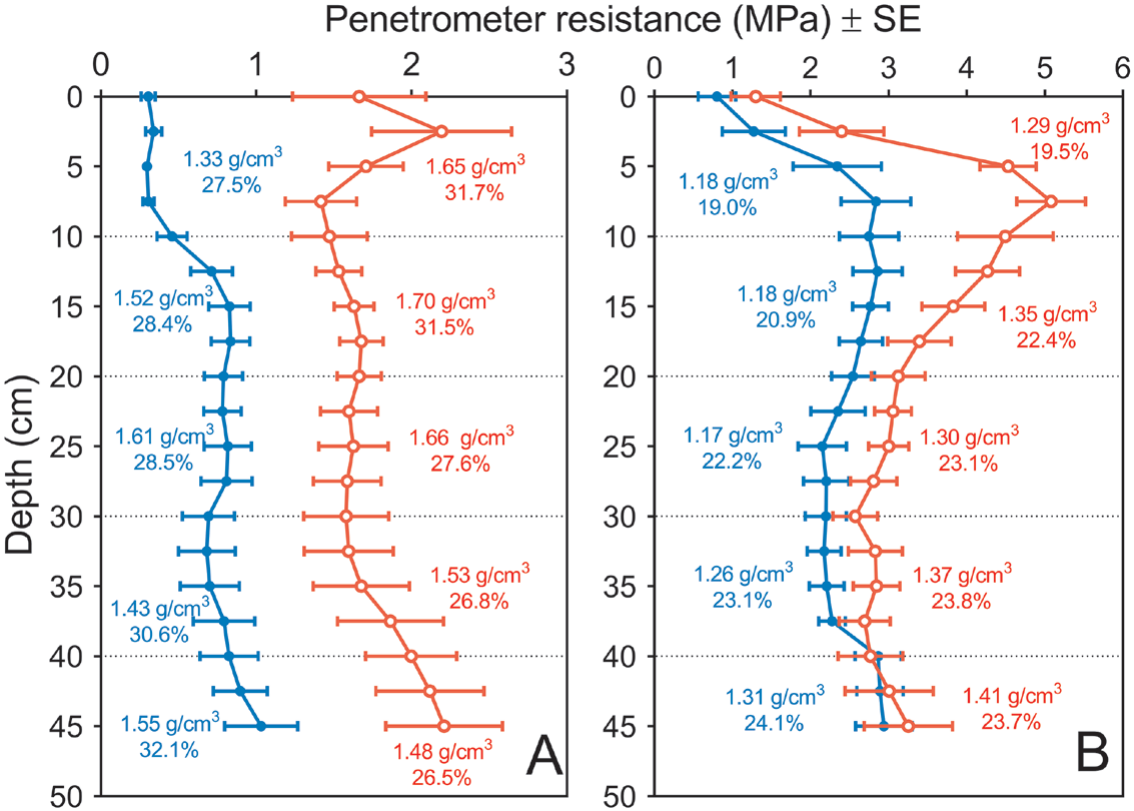
Penetrometer resistances ±SEs as measured by a penetrometer for compacted (red) versus non-compacted (blue) treatments at (A) the Apache Root Biology Centre and (B) the Russell E. Larson Agricultural Research Center–Pennsylvania State University field sites before planting. Bulk density and soil moisture content (v/v) were measured within 10 cm depth increments over a 50 cm deep soil profile. Measurements of volumetric moisture content and bulk density with each 10 cm soil depth (non-compacted in blue, compacted in red) are shown in the graph next to the corresponding penetrometer measurements.

The first field site was planted on 16 June 2016 at the Apache Root Biology Center (hereafter referred to as ARBC), Willcox, AZ, USA (32°01'N, 109°41'W) where the soil is a Grabe series (coarse-loamy, mixed, superactive, calcareous thermic Torrifluvent) clay loam. The second field site was planted on 10 July 2017 at the Russell E. Larson Agricultural Research Center in Rock Springs (hereafter referred to as PSU), PA, USA (40°42'N, 77°57'W) on a Hagerstown series silt loam soil (fine, mixed, semi-active, mesic Typic Hapludalf). To increase mechanical impedance, heavy machinery (four-wheel tractor, 4 t at ARBC and three-axle truck, 20 t at PSU) was passed over the treated plots (eight passes at ARBC and four passes at PSU). Penetrometer resistance (Fieldscout SC900 Compaction Meter, 1/2 inch cone, Spectrum Technologies Inc., Aurora, IL, USA), bulk density, and volumetric moisture content through the soil to a depth of 50 cm were measured (Fig. 1). Soil moisture content was monitored at the whole-plot level using PR2/6-tubes (Delta-T Devices Ltd, Cambridge, UK) with measurement at 10, 20, 30, 40, 60, and 100 cm depth at ARBC and the multi-plexed TDR-100 (Time Domain Reflectometer) system (Campbell Scientific Inc., Logan, UT, USA) installed at 15 cm and 30 cm depth at PSU. Irrigation, nutrients, and pesticides were applied as needed (Supplementary Table S1 at *JXB* online). Twelve maize (*Zea mays* L.) recombinant inbred lines (genotypes) were selected from a pre-screen of the 24 genotypes used by Chimungu *et al.* (2015). The genotypes differed in root anatomical phenotypes and soil penetration ability. These genotypes were planted in a completely randomized split plot design after the application of the compaction treatment, with treatments (compaction and non-compaction) at whole-plot levels and genotypes on subplot levels for both field sites. Each genotype was planted within four row subplots (3.05 m×4.57 m) within each whole plot and individual plants were spaced 23 cm apart within a row and 76 cm between rows.

### Rooting depth

Soils were cored at tasselling which was 55 d and 51 d after planting for ARBC and PSU, respectively. A coring tube fitted with a 4.5 cm diameter, 60 cm long plastic sleeve was driven into the soil (Trachsel *et al.*, 2013) for assessment of rooting depth. In combination with the increased penetrometer resistance at the 10–35 cm depth (or 0–45 cm in the case of ARBC) for compacted fields, the root system was considered sufficiently sampled by the maximal achievable sampling depth of 60 cm. Cores were stored at 4 ºC, divided into 10 cm increments, and roots were washed free of soil over a 800 µm soil sieve. Washed roots were spread on a glass tray filled with water and analysed with WinRHIZO Pro 2013e software (Regent Systems Inc., Quebec, Canada). All images were taken at 400 dpi (15.75 pixels mm^–1^) resolution, dust removal set at high, and no speed priority selected. To assess the capability of roots to grow through an impeded zone, we focused on the coarse root fraction (>1 mm diameter) rather than fine roots (<1 mm diameter). Root diameter classes were set at 0.5 mm increments up to 4.5 mm in order to allow for coarse root length (>1.0 mm diameter) calculations. The rooting depth (D_75_) above which 75% of the coarse root length can be found within the 60 cm soil core was estimated by linear interpolation (Schenk and Jackson, 2002). Total root length (TRL) and 75% of the total root length (TRL_75_) measurements within the 60 cm core were first calculated from the cores. Next, cumulative measurements of root length were calculated for each 10 cm coring increment to determine the coring segment (the segment for which TRL_75_ was between the cumulative boundary measures) for which the D_75_ was to be linearly interpolated.

### Plant harvest, anatomical sampling, and image analysis

**Fig. 2.**
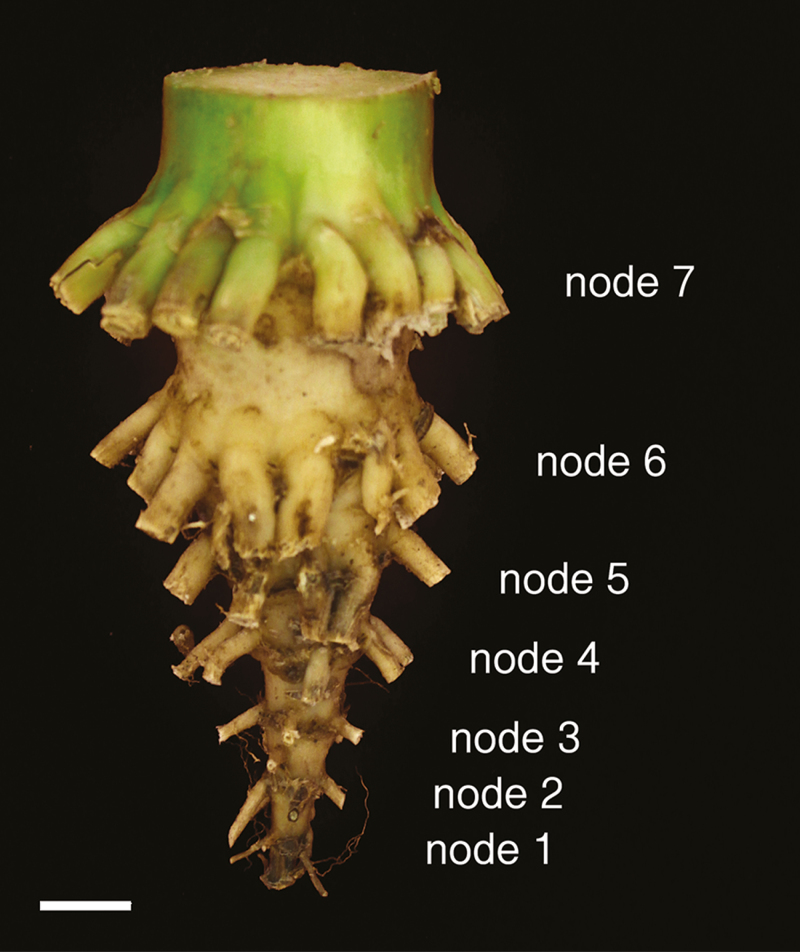
Root crown with the roots clipped off to the base to reveal the different nodal tiers. Each tier of nodal roots is labelled. Scale bar=1 cm. The image was provided by Larry York.

Two plants per subplot (four replicate subplots per compaction treatment) were selected and sampled by ‘shovelomics’ (Trachsel *et al.*, 2011), and measurements within each subplot were averaged, yielding four replicates per genotype–compaction treatment combination. Shoots were dried for several days at 60 °C. Crown and brace roots were imaged to verify root angles between genotypes within field site and treatment (York *et al.*, 2015), as root angle could influence D_75_. Images were analysed with ImageJ and root angles were recorded (Supplementary Table S2; Supplementary Fig. S1). Roots of nodes 3 and 4 from each plant (two plants per subplot) were selected for anatomical analysis and sampled 30 mm from the crown base (Fig. 2). Approximately 30 mm long sections of roots were stored in 75% ethanol in water (v/v). Roots from nodes 3 and 4 were selected as these would contribute most to vegetative growth. Nodes 1 and 2 are very small and more closely associated with a plant at the seedling stage. Nodes 5 and 6 were not selected as these form later in plant development. Additionally nodes 5 and 6 can be formed as brace roots, where they emerge aboveground and do not penetrate deeply at tasselling.

**Fig. 3.**
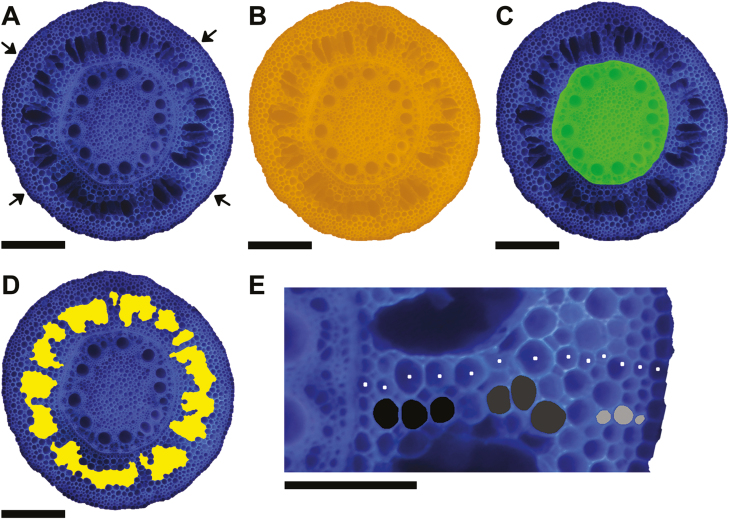
Illustration of the different anatomical traits that were directly measured. (A) Original cross-sectional image of a root obtained by laser ablation tomography (LAT). (B) Root cross-sectional area (RCSA) is indicated in orange and (C) total stele area (TSA) is indicated in green. (D) Aerenchyma area is indicated in yellow. (E) One of four places where inner (black), middle (dark grey), and outer (light grey) cell area were measured as well as the cell file number (white dots). Arrows in (A) indicate that measurements of (E) were taken from four different places around the cross-section. From (B) and (C), total cortical area (TCA) can be calculated and, together with the aerenchyma area measured in (D), the non-aerenchyma area in the cortex can be calculated. From (B–D), relative measures of cortex to stele ratio (TCA/TSA), cortex to cross-sectional area ratio (TCA/RCSA), stele to cross-sectional area ratio (TSA/RCSA), cortex taken up by aerenchyma ratio (AA/TCA), and cross-sectional area taken up by aerenchyma ratio (AA/RCSA) can be calculated. Scale bars (A–D)=500 µm, scale bar (E)=200 µm.

LAT (Hall *et al.*, 2019; Strock *et al.*, 2019) was used to obtain cross-sectional images (Fig. 3A). LAT uses a nanosecond pulsed laser beam (Avia 7000, 355 nm pulse, Coherent, Santa Clara, CA, USA) focused into a single-line scanning beam with a HurryScan 10 galvanometer (Scanlab, Puchheim, Germany) to vaporize and sublimate tissue in front of a Canon T3i camera fitted with a ×5 micro lens (MP-E 65 mm) focused on the ablation plane. Samples were guided into the ablation plane by a three-axis motorized stage (ATS100-100, Aerotech Inc, Pittsburgh, PA, USA) at a speed of 30 µm s^–1^. Images for anatomical assessment were obtained from the central region of the 30 mm sampled sections between laterals. One image was obtained per sampled root section and anatomical traits were averaged per node across the two plants sampled per subplot.

A root cross-section is constructed of diverse tissues, and each tissue trait can be explained as a combination of cellular traits and/or other tissue-related traits. For instance, cellular traits such as CF and inner (IN), middle (MID), and outer (OUT) cortical cell area, measured on cells, from three cell layers, at the inner, middle, and outer regions of the cortex, respectively, build up the cortical tissue. Differentiation between the outer and inner cortex regions has been made (Veen, 1982; Baluška *et al.*, 1993; Striker *et al.*, 2007), while differentiation between the inner and middle cortical region should be made as aerenchyma formation starts in the middle of the cortex (Campbell and Drew, 1983). Similar differentiations between cortical regions have been made by Burton *et al.* (2013*b*) and Chimungu *et al.* (2015). Aerenchyma-related traits can be considered as tissue traits as their dimensions are more closely related to that of tissues than those of cells; moreover, aerenchyma also has tissue functions related to it. To measure or calculate these cellular and tissue-related traits, four different object directories were created in objectJ (Vischer and Nastase, 2009), a plugin for Fiji/ImageJ (Schindelin *et al.*, 2012) over a root cross-section image (Fig. 3B–E shows a representation of all the directly measured traits).

### Statistical analysis

Statistical analysis and visualizations were carried out in Graphpad Prism version 7.04 (Graphpad Software, La Jolla, CA, USA) or R version 3.5.0 (R Core Team, 2018). A bivariate approach was used to identify outliers on the basis of the RCSA data within their respective compaction treatment×genotype×node combinations, as outliers for other anatomical data were linked to outliers for RCSA data. Outliers were replaced by a single observation per subplot instead of the average observation of two plants per subplot in the case where it was clear that the sample did not represent the correct node (this related to ~1% of the data). Principal component analysis (PCA) was carried out to elucidate the anatomical trait relationships over different nodes, treatments, and field sites. Principal components (PCs) were retained on the basis of eigenvalues >1. Split-plot analysis with treatment on a whole-plot level and genotype on a subplot level was carried out within node and field site combinations to assess the effects of compaction treatment and genotype on RCSA and the total cortical area (TCA)/RCSA ratio. Allometric relationships were assessed by fitting a linear regression model on the natural log of the anatomical trait against the natural log of shoot biomass. Based on TCA/RCSA, RCSA, and allometry observations, genotypes were classified as thickening and non-thickening for node 3. A generalized linear model was used to investigate the effect of genotype, field site, root class, treatment, and thickening on D_75_. Thickening was represented by TCA/RCSA data that were Box-Cox transformed for normality prior to running a general linear model with gamma distribution for D_75_. An analysis of covariance (ANCOVA) was used to investigate the interaction effects between the factors thickening, field site, and compaction treatment. A second set of PCAs on anatomy variables in relation to D_75_ within each node and within compacted treatments were performed. Pearson correlations between D_75_ and anatomical traits for each node were used to select independent variables for building multiple regression models. Stepwise multiple regression was carried out to describe a model based on all the anatomical traits. To further understand if either cellular or tissue-related traits contribute to rooting depth D_75_, additional models were constructed by selecting traits on either the tissue or cellular level; after which models were compared with each other. Variance inflation factors were calculated to inspect multicollinearity (Miles and Shevlin, 2001), and the Akaike information criterion identified the best fitting model (Konishi and Kitagawa, 2008).

## Results

### Cellular and tissue-related trait relationships

**Fig. 4.**
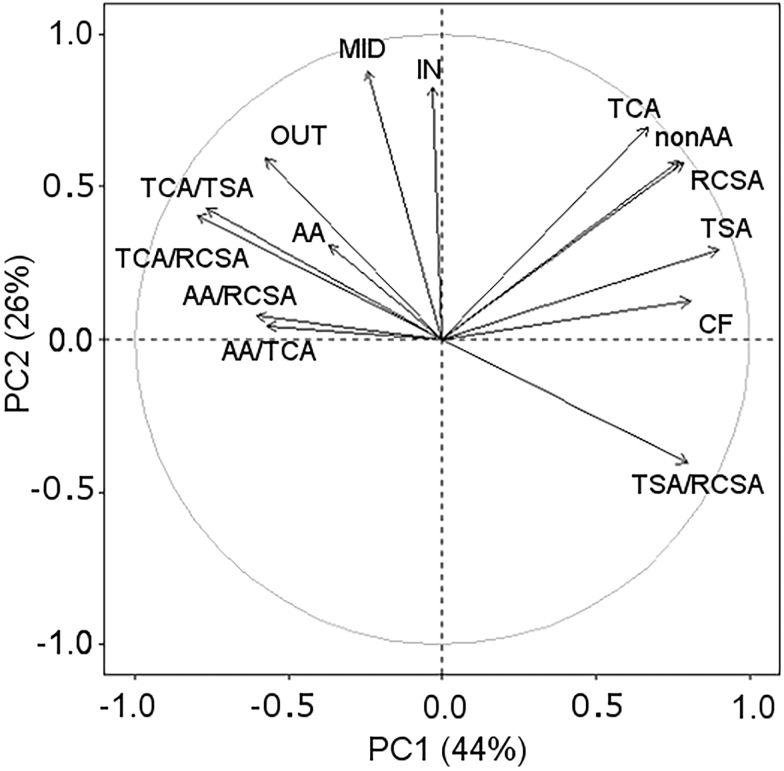
Principal component analysis (PCA) of 14 anatomical traits from root cross-sections across different fields, compaction treatments, and nodes. PCA loadings of the different variables illustrate how different anatomical traits relate to each other. Traits with arrows that group together are correlated to each other; traits with arrows in the opposite direction are negatively correlated with each other. When variables appear orthogonally from each other, associated traits do not correlate. Length of the arrow illustrates how strongly the trait is associated with each PC. Clockwise, abbreviations stand for: TCA, total cortical area; nonAA, non-aerenchyma cortical area; RCSA, root cross-sectional area, TSA, total stele area, CF, cell file number; TSA/RCSA; ratio of stele to root cross-sectional area, AA/TCA, ratio of cortex taken up by aerenchyma; AA/RCSA; ratio of cross-sectional area taken up by aerenchyma; TCA/RCSA, ratio of the cortex to root cross-sectional area; TCA/TSA, ratio of the cortex area to the stele area; AA, aerenchyma area; OUT; cell area of cells in the outer cortical region; MID. cell area of cells in the middle cortical region; IN, cell area of cells in the inner cortical region.

Trait variation was observed within and across nodes as well as field sites (Supplementatry Fig. S2) and among genotypes (Table 1). In the PCA (Fig. 4), the three retained dimensions explained 88% of the total variation in root anatomy. Root tissue traits, total cortical aerenchyma, non-aerenchyma cortical area, and total stele area (TSA) were more closely related to RCSA than cellular traits. Non-aerenchyma cortical area and total cortical area (TCA) explained the RCSA better than the TSA. Cellular traits of MID, IN, and OUT cortical cell area correlated to each other, while IN and MID were more closely related to each other than to OUT. All cell area traits were orthogonally oriented from the CF, indicating no correlation between cell area and the number of cell layers. TCA and RCSA were correlated to both dimensions and that was due to the fact that traits such as cell file layer versus IN and MID were found on different PCs. Although cell file layer was not correlated with IN and MID, all these traits were correlated with the cortex, which in turn was related to RCSA. Interestingly, the cellular area trait OUT was orthogonally oriented versus RCSA; this meant that OUT was not related to RCSA or TCA. OUT cells were smaller than MID cells, but IN cells were similarly small (Supplementary Fig. S2) and still contributed to RCSA. Aerenchyma traits were closely correlated with PC3, with the exception of non-aerenchyma cortical area, and therefore was independent from the other cellular or tissue traits.

**Table 1.** Summary of split-plot ANOVA (*F* ratios) on the root cross-sectional area (RCSA) and the cortex to cross-sectional area ratio (TCA/RCSA)

Effect	RCSA								TCA/RCSA							
	Node 3				Node 4				Node 3				Node 4			
	ARBC		PSU		ARBC		PSU		ARBC		PSU		ARBC		PSU	
Compaction treatment	8.54	*	2.52		4.43	°	2.70		19.54	**	12.29	***	9.29	**	2.31	
Genotype	1.62		3.42	***	1.93	*	6.01	***	8.87	***	2.13	*	7.87	***	5.21	***
Compaction treatment×genotype	1.27		1.60		1.59		2.38	*	1.84	°	0.95		1.03		1.08	

Treatment (compacted versus non-compacted plots) is shown on a whole-plot level and genotype (12 genotypes) on a subplot level for root sections. Data are across two different nodes and from two different field sites: the Apache Root Biology Center (ARBC) and Pennsylvania State University (PSU). °significance level at *P*≤0.1, *significance level at *P*≤0.05, **significance level at *P*≤0.01, ***significance level at *P*≤0.001.

### Anatomical traits are dynamic and dependent on field site, node, and compaction

**Fig. 5.**
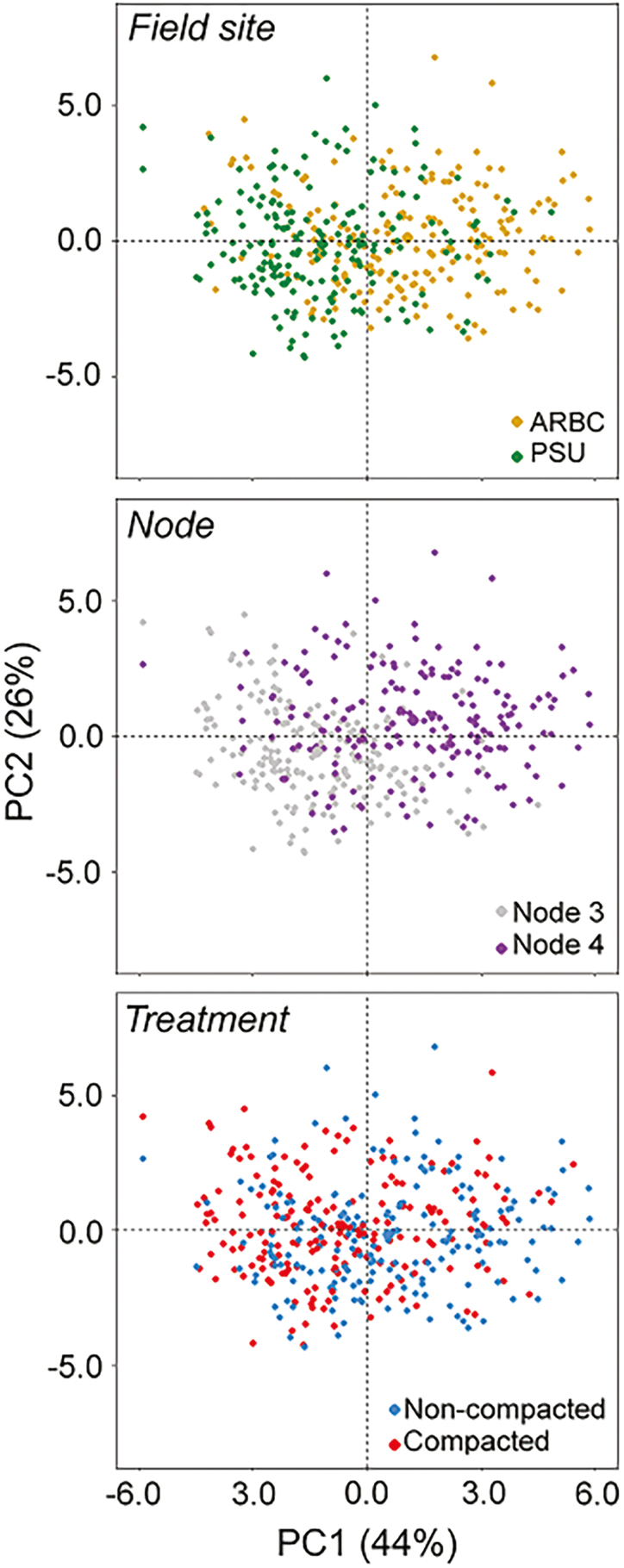
Principal component scores of anatomical data on PC1 and PC2. Data can be visualized for each field site, node, and treatment, showing that anatomical traits are dependent on field site, node, and compaction.

The effects of field site, node, and compaction were visualized by colouring the PCA scores (Fig. 5). Visualization of the first and second PCs shows that field sites and nodal data cause separation, while treatment had more overlap. More overlap of the point clouds was seen in the second versus third dimension projections as compared with the first versus second dimension projection. This means that for traits such as aerenchyma, effects of field site and node were smaller than the effects of node and field site on CF.

### Genotypic and treatment effects on root cross-sectional area and cortical tissue ratios

**Fig. 6.**
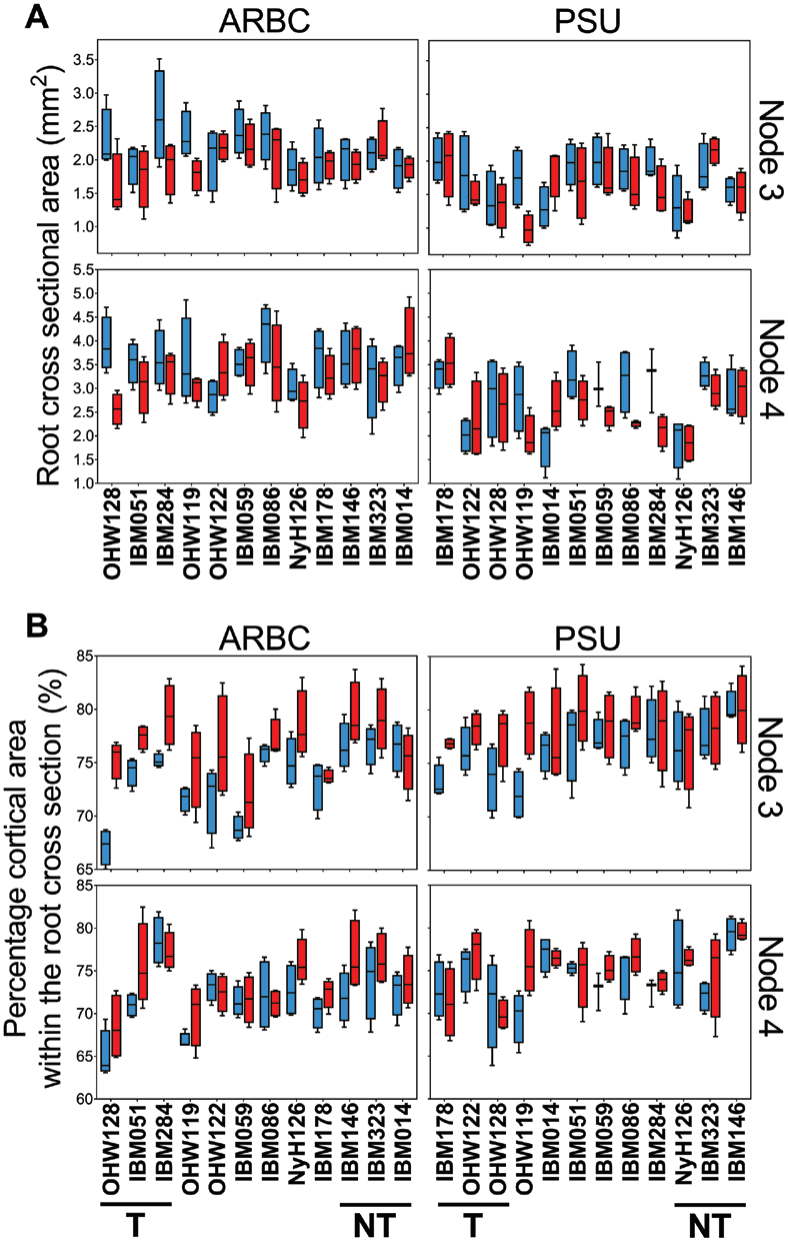
Effects of impedance on root anatomical traits. (A) Boxplots showing the root cross-sectional area (mm^2^) and (B) the percentage of root cross-section that is cortical area (%). Data per graph are split up over different nodes and over different field sites (ARBC or PSU) and visualized per genotype. Compacted data in red; non-compacted data in blue. Thickening and non-thickening selected genotypes are identified by T and NT, respectively.

Genotype had a significant effect on both RCSA and TCA/RCSA across both nodes, with the exception of RCSA for node 3 at ARBC (Table 1). We did not observe thickening as an increase of RCSA under compaction; moreover, RCSA was significantly negatively affected by treatment at ARBC (Table 1; Fig. 6A). The effect of genotype within compaction or non-compaction on the RCSA is shown in Supplementary Fig. S3. Thickening of cortical tissue measured as TCA/RCSA was responsive to the compaction treatment as well as to genotype (Table 1; Fig. 6B) as TCA/RCSA increased under all but one node–field combination (node 4–PSU) (Table 1). The different genotypes within compacted or non-compacted conditions are shown in Supplementary Fig. S4. Under compaction, the mean TCA/RCSA value was greater but non-significantly different from non-compacted conditions for most genotypes at both sites (Fig. 6B). Roots had greater cortical expansion in response to the compaction treatment in node 3 versus node 4 in general (Fig. 6B).

### Node-specific allometry affects root anatomy

**Fig. 7.**
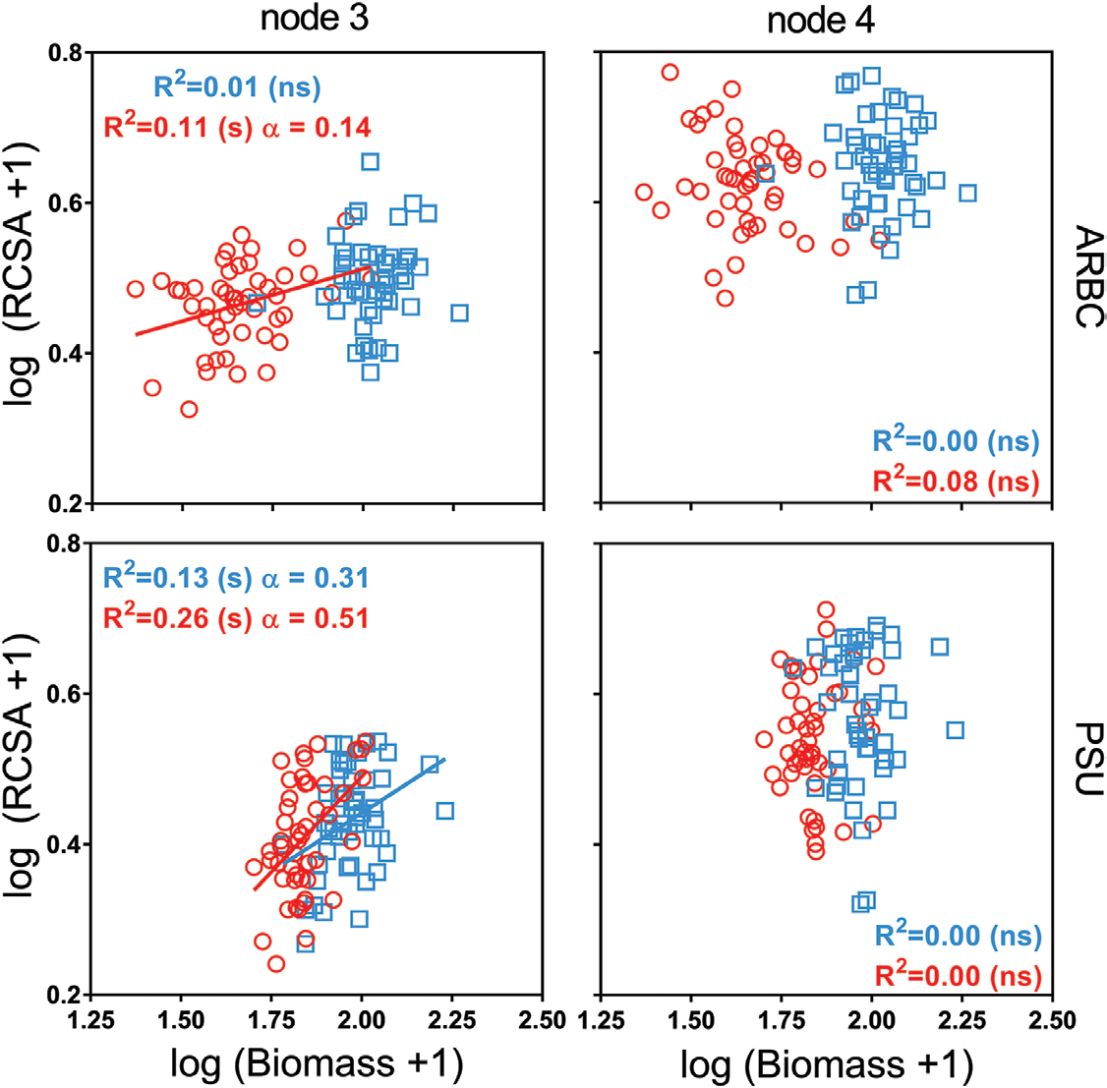
Allometric relationships within nodes 3 and 4 under compacted (red circles) and non-compacted (blue squares) conditions. Full lines indicate that allometry is present. No significant allometric relationship is found under node 4, while node 3 root cross-sectional area scales allometrically across field sites for compacted roots. The allometric scaling component (α) depicts allometry when the relationship is significant (*P≤0.05, ***P≤0.001), ns stands for non-significant.

Biomass did not differ between genotypes within the compaction or non-compaction plots (Supplementary Fig. S5). Soil compaction reduced shoot biomass when comparing compacted and non-compacted plots (Fig. 7); therefore, allometry or proportional growth should be factored into the analysis. For RCSA, allometric relationships were dependent on nodal position as allometric effects were only observed for node 3 and across both field sites (Fig. 7). Node 3 RCSA was hypoallometric, as the scaling component α was <1. Under compaction, plants that had a greater biomass formed greater RCSA for node 3 roots. As allometry could have obscured the thickening effect, as we saw smaller RCSA than expected, relative measures of TCA/RCSA identified thickening better. No allometric effects were observed for node 4.

### Non-thickening versus thickening genotypes and their relationship to rooting depth

**Fig. 8.**
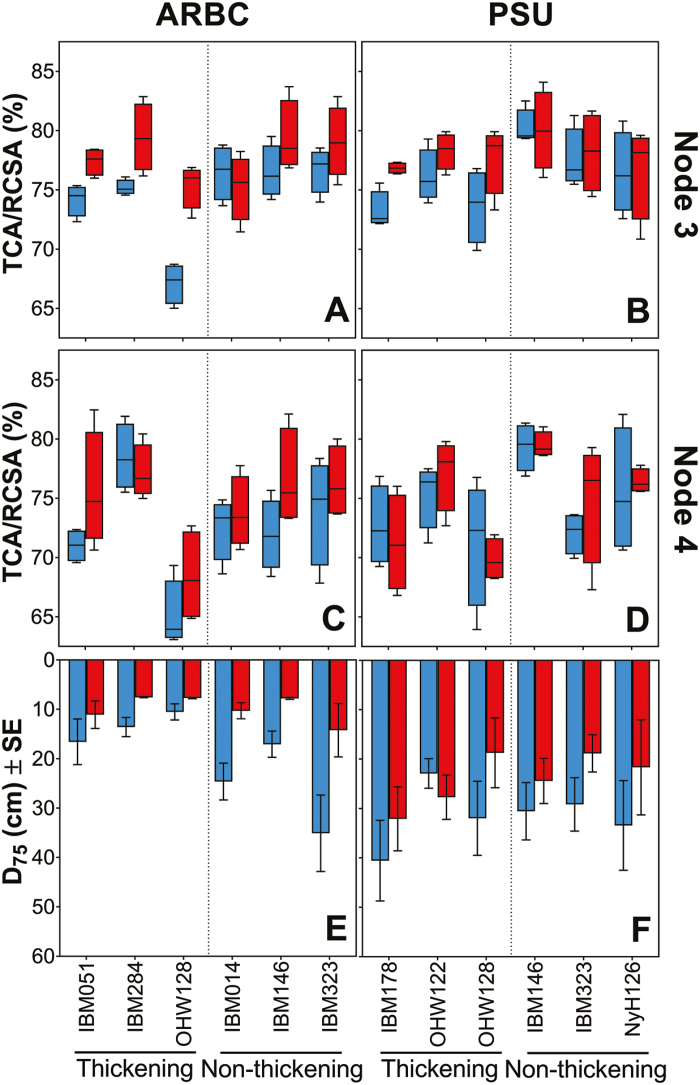
Changes of the percentage of cortical area (TCA/RCSA) and related rooting depth (D_75_) of thickening and non-thickening genotypes across nodes and field sites. Compacted data in red; non-compacted data in blue.

Allometry, RCSA, and TCA/RCSA observations were taken into account to identify thickening genotypes. Differences between thickening and non-thickening roots for TCA/RCSA were less clear for node 4 (Fig. 8C, D). Both RCSA and TCA were reduced under compaction. For node 3, non-thickening roots were characterized by a greater TCA/RCSA in general, while thickening roots had a lower TCA/RCSA under non-compacted conditions and increased in TCA/RCSA under compaction (Fig. 8A, B). The general linear model on rooting depth (D_75_) indicated that field site had an effect on rooting depth, and compaction reduced rooting depth (Supplementary Table S3). Interaction effects were present between field site and thickening and between field site and compaction treatment (Supplementary Table S4). Compaction had a greater effect on rooting depth at ARBC than at PSU (Fig. 8E, F). Node 3 roots of some genotypes (IBM051, IBM178, IBM284, OHW122, and OHW128) thickened while node 3 roots of other genotypes (IBM014, IBM146, IBM323, and NyH126) did not (Fig. 8), while some genotypes remained stable in this phenotype at both sites (OHW128, IBM146, and IBM323) and others did not (IBM051, IBM284, IBM014, and NyH126). Root thickening was associated with rooting depth in one field site location (ARBC) under non-compacted conditions (Fig. 8E, F; Supplementary Table S4). At ARBC, non-thickening genotypes grew deeper than thickening genotypes under non-compacted conditions, but under compaction no differences in D_75_ between thickening and non-thickening genotypes were present (Fig. 8E). At PSU, rooting depth was reduced in all but one case (genotype OHW122) by compaction, but no differences were found between thickening and non-thickening genotypes in either compacted or non-compacted treatments (Fig. 8F).

### Node-dependent anatomical traits associated with deeper rooting in compacted soil

**Fig. 9.**
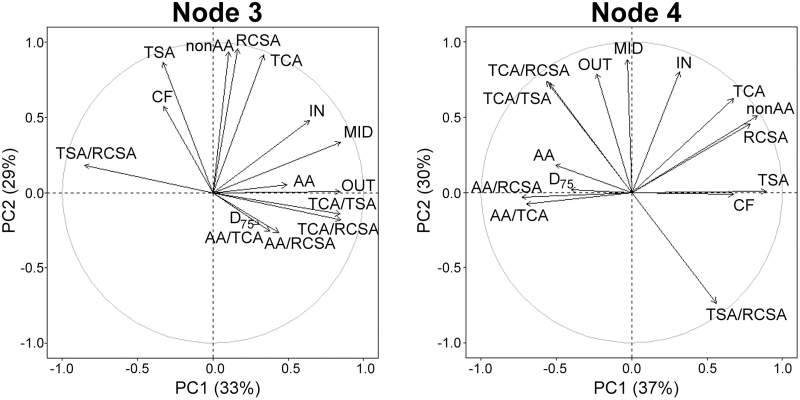
Relationships between anatomical traits and rooting depth D_75_ for two different nodes as analysed by PCA under compaction. Abbreviations for traits can be found in the legend of Fig. 3. The angle between variables represents the correlation between those variables; when the angle is 90°, the variables are not correlated in this dimensional projection.

For each node, for compaction, a PCA was performed on the anatomical data and D_75_ (Fig. 9). For node 3, five PCs were retained, explaining 90% of the data variation. Rooting depth (D_75_) was most associated with PC4. D_75_ was negatively correlated with CF in all PC projections, while other traits were harder to interpret as they contributed to other PCs. For node 4, three PCs were retained, which explained 83% of the data variation. Rooting depth for node 4 was not associated with any of the retained PCs, suggesting that it must have a weaker relationship with anatomy than in node 3.

**Fig. 10.**
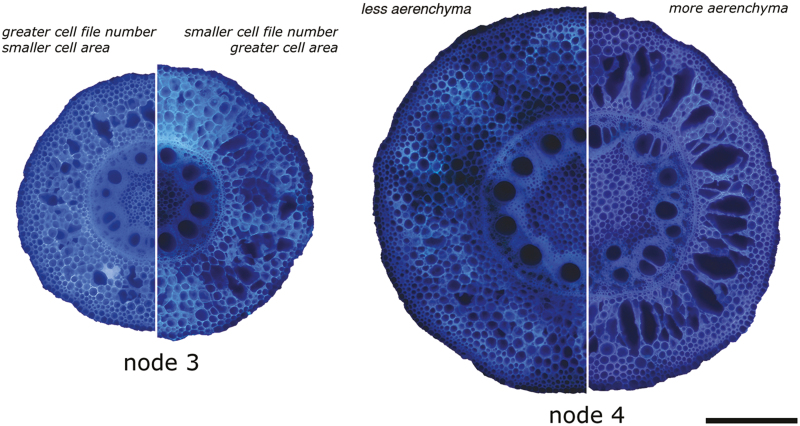
Cross-sectional images obtained by laser ablation tomography depicting the anatomical differences within each node. Half of each node represents a different anatomical make-up. Node 3 cross-sections with greater cell file number, smaller cells on the left of node 3, and smaller cell file number and larger cells on the right of node 3. Node 4 cross-sections with lower aerenchyma area on the left of node 4 and greater aerenchyma area on the right of node 4. All images were taken under compacted conditions. Scale bar=500 µm for all images.

D_75_ was negatively correlated with CF number and positively correlated with MID, OUT, aerenchyma area (AA) and AA/RCSA for node 3 (Supplementary Table S5). For node 4, D_75_ was negatively correlated with RCSA, TSA, non-AA, and CF, and positively correlated with AA, AA/TCA, and AA/RCSA. Summaries for each individual multiple regression are shown in Supplementary Table S6 (node 3) and Supplementary Table S7 (node 4). Multiple regression models are compared in Table 3. Cellular traits (model 5; adjusted *R*^2^=0.19) were better predictors for D_75_ than tissue-related traits (model 3; adjusted *R*^2^=0.04) for node 3 (Table 2A). The best fitting model (lowest Aikake information criterion) was model 2, which included two cellular variables, CF and MID, explaining 20% of the variability in D_75_ (Tables 2A, 3A; *P*<0.001). Therefore, the contribution of cellular traits in node 3 to deeper rooting was significant. Node 3 root sections that contained fewer cellular layers in combination with greater MID cellular area rooted deeper (Fig 10A). For node 4, tissue traits (model 2; adjusted *R*^2^=0.12) were better predictors for D_75_ than cellular traits (model 5; adjusted *R*^2^=0.06) (Table 2B). D_75_ for node 4 roots was negatively influenced by TSA and positively by AA/RCSA (Fig 10B). The model with the lowest Aikake information criterion was model 2, explaining 14% of the variability in D_75_ (Tables 2B, 3B; *P*<0.01). These traits therefore made a small but significant contribution to deeper rooting under impeded conditions, but less so than for node 3. Figure 10 illustrates these differences in anatomical traits across nodes.

**Table 2.** Comparison of different multiple regression models run on (A) node 3 and (B) node 4 data; traits selected on the basis of Pearson correlation with the response variable D_75_.

A	Model		*R* ^2^	Adjusted *R*^2^	*P*-value		AIC	
	1	D_75_~CF+MID+OUT+AA+AA/RCSA	0.24	0.19	4.85E-03	**	662.55	
	**2**	**D**_**75**_ **~CF+MID**	**0.20**	**0.19**	**6.65E-05**	**	**660.02**	
	3	D_75_~AA+AA/RCSA	0.06	0.03	0.09	ns	675.00	
	4	D_75_~AA	0.06	0.04	2.84E-02	*	673.01	
	5	D_75_~CF+MID+OUT	0.22	0.19	1.08E-04	***	660.11	
	6	=model 2						
**B**	**Model**		**R** ^**2**^	**Adj. R** ^**2**^	***P*-value**		**AIC**	
	1	D_75_~RCSA+TSA+CF+AA+AA/TCA+AA/RCSA+non-AA	0.18	0.10	0.03	*	673.10	(m)
	**2**	**D**_**75**_ **~ TSA+AA/RCSA**	**0.14**	**0.12**	**1.83E-03**	*	**666.89**	
	3	D_75_~RCSA+TSA+AA+AA/TCA+AA/RCSA+non-AA	0.17	0.10	0.02	*	672.08	(m)
	4	= model 2						
	5	D_75_~CF	0.07	0.06	1.44E-02	*	671.78	

AIC, Akaike information criterion, with in bold the model with the lowest value for the AIC which represents the best model fitted out of the models tested. Abbreviations for the anatomical traits are given in the legend of Fig. 3. D_75_ stands for the rooting depth where 75% of the total coarse root length within a core can be found. (m) indicates that the model has a multicollinear component. ns stands for non-significant, *significance level at *P*≤0.05, **significance level at *P*≤0.01, ***significance level at *P*≤0.001.

**Table 3.** Summary of multiple regression with the lowest Akaike information criterion values for (A) node 3 and (B) node 4

A	Model 2: D_75_~CF+MID					
		Estimate	SE	*t*-value	*P*-value	
	(Intercept)	43.96	11.36	3.87	2.14E-04	***
	CF	–3.13	0.89	-3.52	6.93E-03	***
	MID	4.67E-03	2.22E-03	2.10	3.88E-02	*
	Multiple *R*^2^	0.205				
	Adjusted *R*^2^	0.186				
	*P*-value	6.65E-05	***			
**B**	**Model 2: D**_**75**_ **~TSA+AA/TCA**					
		Estimate	SE	*t*-value	*P*-value	
	(Intercept)	20.11	4.95	4.06	1.09E-04	***
	TSA	-9.00	5.18	–1.74	8.61E-02	
	AA/TCA	27.46	11.12	2.47	1.56E-02	*
	Multiple *R*^2^	0.14				
	Adjusted *R*^2^	0.12				
	*P*-value	1.83E-03	**			

***level of significance at *P*≤0.001 and **level of significance at *P*≤0.01. Abbreviations for the anatomical traits are given in the legend of Fig. 3. D_75_ stands for the rooting depth where 75% of the total coarse root length within a core can be found.

## Discussion

Root anatomical phenotypes are dynamic, responding to genotype, field site, and soil compaction. Root anatomy contributes to the ability for root penetration through compacted soil, but allometry needs to be taken into account for smaller, older roots. Roots that are thicker from the outset, such as those of the younger node 4, had less anatomical response to hard soil than those of node 3. Moreover, although individual anatomical traits play a role in the ability to penetrate hard soil, radial thickening was not one of them. Within node 3, cellular traits such as CF and MID cortical cell area play an important role, while for node 4, increased cortical aerenchyma and a smaller stele area were associated with deeper rooting in compacted soils.

### Traits are highly interactive and adaptive to their local environments

When comparing between field sites, different RCSAs (Fig. 6A) are observed for the same genotype. Differences in RCSA between field sites could be caused by a difference in soil texture as larger root diameters have been observed in soils with greater aggregate size (Logsdon *et al.*, 1987). Greater root diameters would be capable of displacing larger particles and aggregates (Whiteley and Dexter, 1984). As roots are deflected around larger aggregates that cannot be displaced or penetrated, alternating thin and thicker root diameters can be found along a root axis as the level of impedance changes along the root trajectory (Logsdon *et al.*, 1987), confirming that the arrangement of the soil pore network plays a role in root anatomy. Kirby and Bengough (2002) observed that pea roots, grown in a sandy loam soil, can increase their diameter by 60% when grown at a mechanical impedance of 2 MPa versus 0.7 MPa. When grown in clay loam instead of sandy loam soil, root diameter increased less. Tomato root diameter increased in hard loamy sand more than in hard clay loam, illustrating the importance of soil texture (Tracy *et al.*, 2013). The greater sand fraction and less structured soil, in combination with greater differences in penetrometer resistance between compaction and non-compaction treatment, at the ARBC field site could explain why larger diameters under non-compacted conditions are seen in both nodes versus at the PSU field site. As root diameters respond to their local environments, so must the underlying anatomy. Differences in penetrometer resistance were recorded at both PSU and ARBC fields (Fig. 1) and differences in soil structure were observed. However, under compaction, we observed more tortuous, bent, roots with irregularly shaped root sections (Supplementary Fig. S6). Plasticity for root cross-sectional shape falls outside of the scope of this study, but root deformation in response to the local soil structure warrants further investigation.

Anatomical traits are strongly intercorrelated in similar ways across nodes (Figs 4, 9, and as seen by Yang *et al.*, 2019). Nonetheless anatomy makes a significant contribution to deeper rooting (Table 3). Under specific field and compaction conditions, root anatomy changes (Figs 5, 6). Most interesting are the shifts in tissue ratios between the cortex and stele and changes in aerenchyma, which point to an effect of compaction treatment on the cortex. Here we illustrate that cortical tissues expand (Fig. 6) under compacted conditions. Greater anatomical changes were observed when the differences in penetrometer resistance were greater between non-compacted and compacted plots, as was the case at ARBC (Figs 6, 8; Supplementary Fig. S2). Different phenotypic adaptations were observed with node and genotypic dependence as some node 3 roots thickened, while other did not (Fig. 8). This shows that root systems are highly adaptable across genotypes but also within individual plants. The response to impedance of different nodes and genotypes was non-uniform. Future work might consider these patterns on a larger set of genotypes and under varying levels of mechanical impedance.

### Thickening is node dependent and obscured by allometry

Root thickening is commonly observed in response to mechanical impedance for different plant species and root types in different experimental conditions (Barraclough and Weir, 1988; Materechera *et al.*, 1991; Atwell, 1993; Iijima *et al.*, 2000; Kirby and Bengough, 2002; Colombi and Walter, 2016). Root thickening is often considered beneficial since thicker roots would be less likely to buckle (Whiteley *et al.*, 1982) and would reduce stress at the root tip (Abdalla *et al.*, 1969). Additionally, the elongation zone of impeded roots becomes shorter and moves closer to the root tip and, as such, the friction upon this zone is reduced (Atwell, 1993). On the other hand, greater penetration resistances increased root diameters and energy costs for root elongation for wheat primary roots (Colombi *et al.*, 2019). We observed no direct thickening in our field studies, in contrast to other studies. This could partly be due to the remote measurement of the RCSA near the root crown in the shallower part of the soil. RCSA was not significantly greater in compacted plots (Fig. 6), but TCA/RCSA was greater under compaction of node 3 and to a lesser extent in node 4. Together with the results found in Table 1, it can be concluded that root cortical tissues do react when impeded and that this is genotype dependent.

Soil compaction reduced plant biomass in both field sites (Fig. 7). Shoot growth is coordinated with root growth, and nodes develop in acropetal tiers, becoming progressively thicker in younger nodes. Impedance causes allometric effects in node 3 across field sites but not for node 4; RCSA is therefore more strongly linked to plant size during early growth. Allometric effects on maize root anatomy have been reported previously (Burton *et al.*, 2013*a*). The cortex reacts to compaction and increases in relative size. We observed that the thickening effect had been obscured by an allometric effect in node 3. For node 4, no allometric effect was present (Fig. 7); additionally, no differences in RCSA were caused by compaction (Fig. 6A) and only a significant increase in TCA/RCSA at ARBC was observed (Fig. 6B; Table 1). Therefore, it can be concluded that thickening does not occur in the younger node 4 roots. As greater diameter roots have previously been found to be more capable of growing under compacted conditions [monocotyledons versus dicotyledons (Materechera *et al.*, 1991) and pea versus barley (Stirzaker *et al.*, 1996)], this could also be the case for the younger, thicker roots of maize within the same root system. The difference in RCSA of node 3 and 4 roots (1.16 mm^2^) was only slightly smaller than the difference calculated from the reported diameters of pea and barley from Stirzaker *et al.* (1996). Most studies observing root thickening have done so on seedling roots which are generally small. We studied maize, which has larger roots than small grains (e.g. wheat, barley). Thinner roots or seedling roots might thicken to a greater extent in comparison with roots from older maize plants (younger, thicker nodes) that already have a certain diameter. Thickening might not be present for node 4 roots, due to roots developing as the plant matures being more structural in the support of aboveground biomass, while node 3 roots would be more dependent on anatomical changes at the cellular level in order to grow through impeded zones.

Cortical expansion has been linked to thickening through the increase of CF and/or cell expansion in the radial plane; however, the literature is inconsistent as to the main driver of radial expansion. Maize has been observed to increase cortical cell area, which in turn increases root diameter, when grown in glass beads under pressure, but that study did not consider the effect of additional cell files (Veen, 1982). Iijima *et al.* (2007) found that maize seminal root diameter increased by 80% in response to mechanical impedance, while the cortical thickness increased by 110%. This study also reported a 20% increase in the number of cortical cell tiers under impeded conditions. Cellular area was not measured, but a clear increase in cellular area can be observed from their images (fig. 4 in Iijima *et al.*, 2007). Additionally, we considered different nodal root classes instead of seminal roots in our study. We are not aware of another study investigating root anatomy according to their specific nodes under impedance.

Thickening versus non-thickening genotypes were distinguished based on node 3 (Fig. 8A, B). Both thickening and non-thickening genotypes show similar rooting depths on compacted plots (Fig. 8E, F). Root thickening was negatively associated with rooting depth growing in non-compacted ARBC field conditions (Fig 8E; Supplementary Table S4). Different nodal tiers are present within the same plant, with increasingly steeper root angle with each node formed (Araki *et al.*, 2000; Wu *et al.*, 2015; York *et al.*, 2015). As younger nodes are innately thicker, it could be that these roots experience less impedance stress than the older thinner nodes. Less root thickening would occur for these younger nodes, which supports the view that non-plasticity for thickening would be better for growing through impedance. Rooting depth was also influenced by the field site and compaction (Supplementary Tables S3, S4). Site differences including growth conditions, weather, and soil physical characteristics such as soil texture and structure could have influenced root growth.

### Reduced cell file number is an important cellular trait under compaction

CF and cell area (OUT, MID, and IN) variables were found on different dimensions in all PCA results (Figs 4, 9) and are independent from each other. A similar result has been observed under nitrogen stress; under low nitrogen, cortical cell area was reduced but CF changed little (Yang *et al.*, 2019). This evidence suggests that CF and cell area traits are independent. We found that reduced CF is an important trait when growing in compacted plots (Tables 2, 3). For node 3, we see this manifested in combination with the addition of MID in the model (Table 3). Greater MID in combination with reduced CF (Fig. 10A) was positively associated with increased rooting depth for node 3 roots.

Within the cortex, different cell layers react differently to mechanical impedance. In barley, cell diameters of the outer cortical cells increase under mechanical impedance, while inner cell diameters become smaller (Wilson *et al.*, 1977), with smaller cells shown to be more rigid and strong in maize (Chimungu *et al.*, 2015). In our study, we show that OUT increases, but in comparison with MID remains small, as do the inner cell layers (Supplementary Fig. S2). Considering cortical attributes, Chimungu *et al.* (2015) proposed a root anatomical ideotype that would facilitate penetration of hard subsoils. The outer protective layer of the cortex should consist of small cells to counteract bending and buckling in combination with larger cortical cells in the inner layers that contribute to a larger diameter and reduced metabolic cost. Barley roots under moderate mechanical impedance increased in diameter, while the tensile strength of those roots remained unaffected (Loades *et al.*, 2013). Further research is needed to link the effect of changing root anatomical characteristics to the physical properties of roots.

Assuming RCSA is either built out of larger cell areas with fewer cell files or smaller cell areas with more cell files, the greater CF would entail additional metabolic costs (Lynch, 2015). It has been proposed that reduction of metabolic cost leads to deeper rooting (Lynch, 2015; Lynch and Wojciechowski, 2015). Recently, Colombi *et al.* (2019) showed that energy costs for root elongation were increased by mechanical impedance, but that energy costs were reduced for those wheat genotypes with greater cortical cell diameters. The oxygen demand of impeded roots has been observed to be greater than under control conditions, with elongating cells showing higher critical levels of O_2_ pressures of respiration; additionally, diffusion pathways became longer due to radial thickening (Hanbury and Atwell, 2005). Extra cell walls, from increased CF, will contribute to slower O_2_ diffusion across the root and will demand more oxygen by the cortical tissue (Hanbury and Atwell, 2005). In order to produce additional cell file layers in a root, it will also be necessay to adjust its pattern of cell division within its meristem. Within the meristem, cell divisions will occur anticlinally adding cells to a cell file, while periclinal cell division (adding cell file layers) occurs far less (Shishkova *et al.*, 2008; Potocka *et al.*, 2011). This would make the addition of a cell file dependent on meristematic changes. A meristematic change, such as the switch from a closed to open meristem, has been observed in maize under mechanical stress (Potocka *et al.*, 2011), though what this means and how often this has an effect on CF changes remains unclear.

### Increased aerenchyma is an important tissue trait under compaction

The significant effect of cortical aerenchyma (as AA or AA/RCSA) on rooting depth (Tables 2, 3; Fig. 10) can be interpreted as deriving from its effect on oxygen transport in the root (Colmer, 2003) and in the context of the metabolic costs of soil exploration (Lynch, 2013). When soils become stronger due to drying, and associated changes in impedance rise, it has been shown that aerenchyma formation has a positive effect on overall root growth (Zhu *et al.*, 2010; Jaramillo *et al.*, 2013). Root cortical aerenchyma contributes to deeper rooting (Lynch and Wojciechowski, 2015). Aerenchyma reduces respiration (Colmer, 2003; Fan *et al.*, 2003; Chimungu *et al.*, 2015). In contrast to harder, and potentially drier soils, compacted soils have less porosity and greater potential to become waterlogged, thus aerenchyma could counteract hypoxia within the tissue. Large lacunae promote longitudinal oxygen flow through the roots, reduce oxygen metabolism, and enable CO_2_ to vent out of the root tissue (Drew *et al.*, 2000; Colmer, 2003; Karahara *et al.*, 2012), and is adaptive under hypoxic conditions (Kawai *et al.*, 1998; Coudert *et al.*, 2010; Lynch and Wojciechowski, 2015). On the other hand, aerenchyma reduces the radial transport of water and nutrients (Hu *et al.*, 2014). Root porosity, enhanced by aerenchyma formation, can weaken root structure, but, when a dense multiseriate, sclerenchymatic ring of cells is present in the outer cortex, the effect can be reduced (Striker *et al*., 2006, 2007). However, as aerenchyma only develops after root penetration, at a considerable distance from the root tip and elongation zone, it is not likely to have a negative effect on the physical aspect of root penetration into hard soil. As aerenchyma is independent from the other anatomical traits (Figs 4, 9) and clear variation exists (Fig. 10B; Supplementary Fig. S2), it merits attention as a breeding target to improve rooting depth (Lynch and Wojciechowski, 2015).

Our study focused on anatomy; however, other root traits may also contribute to overcoming impedance. The presence of mucilage assists in reducing the friction experienced by the root cap by lubricating the soil–root interface (Iijima *et al*., 2000, 2004). Another trait decreasing friction is that of root cap sloughing (Bengough and McKenzie, 1997; Iijima *et al.*, 2000). The root cap itself helps to overcome impedance (Iijima *et al.*, 2003), whilst root tips characterized by a smaller root tip radius to length ratio will increase root elongation as impedance is overcome more easily by this shape (Colombi *et al.*, 2017). Root tip anchorage can be provided by changing root trajectories, for instance when hitting a layer, as well as the presence of root hairs that appear closer to the root tip under impeded conditions (Bengough *et al.*, 2011; Haling *et al.*, 2013). Root architectural traits, such as steep root angles, will also increase the probability of roots penetrating through layers (Jin *et al.*, 2013). Future research should look at synergies between these traits and root anatomy.

### Conclusions

We observed that root thickening in maize was obscured by an allometric effect in node 3, but that the cortical area clearly expands in reaction to mechanical impedance. However, this effect is lost in subsequent root classes, and thickening was not observed in node 4 roots. As node 4 roots were thicker from emergence, they may be less sensitive to impedance. Cellular traits in younger roots might play less of a role here in comparison with the older, thinner, node 3 roots. Genotypes could be classified as thickening or non-thickening in response to soil compaction, but no differences in rooting depth were observed between these groups. In our conditions, we saw no evidence that thickening of root axes in response to impeded conditions contributed to rooting depth in compacted soil. We have shown that anatomy contributes to deeper rooting, especially for older nodal roots. Within their respective nodes, root anatomical traits, such as reduced cell file number and increased middle cortical area were associated with deeper rooting. Aerenchyma, on the other hand, was more important in node 4. Both reduced cell file number and increased cell size as well as aerenchyma are traits that reduce the metabolic costs of roots growing in compacted soils. Therefore, we suggest that a clear distinction between thicker roots, that have the innate capability to grow under mechanical impedance, and thickening roots, as a reaction to impedance, should be more clearly made. Anatomical traits contribute to the ability of a root system to grow under impeded conditions. Root anatomy should be considered and studied more closely to increase our understanding, and ensure that the screening of cultivars is optimized for the exploration of soils in suboptimal conditions due to the hard soil conditions many plants have to contend with.

## Supplementary data

Supplementary data are available at *JXB* online.

Table S1. Field applications.

Table S2. Average brace and crown root angle for the 12 tested genotypes at the two different field sites.

Table S3. General linear model summary of the effect of the factors season, compaction, genotype, node, and thickening on rooting depth D_75_ of selected thickening and non-thickening genotypes.

Table S4. Summary of ANCOVA for the effect of field site, compaction treatment, and thickening on rooting depth D_75_.

Table S5. Pearson correlations for anatomical traits and D_75_.

Table S6. Summary of stepwise multiple regression models for node 3.

Table S7. Summary of stepwise multiple regression models for node 4.

Figure S1. Relationship between root angle and D_75_.

Figure S2. Histograms for each anatomical trait measured within each field site and node.

Figure S3. Biomass ±SE at both field sites under compacted (red) and non-compacted (blue) conditions for each field site.

Figure S4. Differences between genotypes for the trait root cross-sectional area (RCSA) within each node and genotype combination.

Figure S5. Differences between genotypes for the trait ratio of total cortical area to cross-sectional area (TCA/RCSA) within each node and genotype combination.

Figure S6. Example of an irregularly shaped root section of a root grown under compacted conditions.

eraa165_suppl_Supplementary_File001

## Abbreviations

AA: aerenchyma area
ARBC: Apache Root Biology Center field site
CF: cell file number
IN: cell area of the inner cortical region
LAT: laser ablation tomography
MID: cell area of the middle cortical region
OUT: cell area of the outer cortical region
PC: principal component
PCA: principal component analysis
PSU: Penn State University field site
RCSA: root cross-sectional area
TCA: total cortical area
TSA: total stele area
TRL: total root length
TRL_75_: 75% of the total root length.
