# Supplementary material for: Root anatomical traits contribute to deeper rooting of maize under compacted field conditions
Source: J Exp Bot. 2020 May 18;71(14):4243–57. doi: 10.1093/jxb/eraa165 (PMC7337194; doi:10.1093/jxb/eraa165)
Supplement: eraa165_suppl_Supplementary_File001 [file eraa165_suppl_supplementary_file001.pdf]

Supplementary data

Table S1 – Field applications.

	Field applications					
	Irrigation		Fertilizers		Pesticides	
**ARBC**	18/06/2016	0.94 mm	04/06/2016	ProSol (15 gallons/acre)	17/06/2016	Atrazine and S-metolachlor
	21/06/2016	0.60 mm	14/06/2016	ProSol (12.5 gallons/acre)	14/06/2016	Copper, Azoxystrobin and Chlorantraniliprole
	22/06/2016	1.20 mm	15/06/2016	UAN (38.3 lbs/acre)	23/06/2016	Chlorantraniliprole
	23/06/2016	0.32 mm	16/06/2016	ProSol (12.5 gallons/acre)		
	25/06/2016	1.60 mm				
	28/06/2016	0.30 mm				
	04/06/2016	0.24 mm				
	06/06/2016	0.20 mm				
	08/06/2016	0.50 mm				
	10/06/2016	0.72 mm				
	17/06/2016	0.75 mm				
	20/06/2016	0.50 mm				
	24/06/2016	0.50 mm				
	27/06/2016	0.50 mm				
	09/08/2016	0.50 mm				
**PSU**	*No irrigation applied as moisture content remained stable during growing season*		urea Nitrogen (200 lbs/acre) applied prior to planting		*No pesticides were applied*	

Table S2 – Average brace and crown root angle for the twelve tested genotypes at the two different field sites.

		Crown roots						Brace roots					
		Non-compacted			Compacted			Non-compacted			Compacted		
	Genotype	Average root angle (º) ± SE			Average root angle (º) ± SE			Average root angle (º) ± SE			Average root angle (º) ± SE		
**ARBC**	IBM014	50	±	4	43	±	4	45	±	4	53	±	2
	IBM051	47	±	3	49	±	2	57	±	3	48	±	2
	IBM059	50	±	3	42	±	3	45	±	3	46	±	3
	IBM086	56	±	3	49	±	4	50	±	2	50	±	3
	IBM146	57	±	2	53	±	3	58	±	3	53	±	3
	IBM178	45	±	3	49	±	1	48	±	3	50	±	2
	IBM284	48	±	3	48	±	3	49	±	2	49	±	2
	IBM323	45	±	2	51	±	3	41	±	3	49	±	2
	NyH126	50	±	3	46	±	3	42	±	2	48	±	3
	OHW119	49	±	3	54	±	1	54	±	3	54	±	3
	OHW122	51	±	4	48	±	1	52	±	3	55	±	3
	OHW128	49	±	3	51	±	2	49	±	3	53	±	2
**PSU**	IBM014	44	±	4	45	±	4	47	±	5	45	±	6
	IBM051	54	±	2	53	±	3	45	±	3	51	±	4
	IBM059	59	±	3	45	±	3	34	±	5	31	±	3
	IBM086	56	±	3	48	±	3	42	±	3	44	±	4
	IBM146	49	±	3	56	±	4	48	±	3	48	±	2
	IBM178	46	±	3	47	±	5	45	±	4	52	±	8
	IBM284	49	±	4	50	±	5	38	±	5	37	±	3
	IBM323	55	±	2	52	±	4	33	±	2	37	±	2
	NyH126	56	±	4	54	±	3	47	±	3	37	±	2
	OHW119	40	±	4	50	±	4	50	±	3	57	±	3
	OHW122	39	±	6	46	±	5	45	±	5	61	±	5
	OHW128	57	±	2	50	±	5	43	±	6	52	±	11

Table S3 – General linear model summary of the effect of factors season, compaction, genotype, node and thickening on rooting depth D_{75} of selected thickening and non-thickening genotypes. *** level of significance at p ≤ 0.001.

	Estimate	SE	t-value	p-value	
(Intercept)	0.07	0.02	3.47	6.52E-04	***
Field site	-0.03	4.00E-03	-6.86	1.17E-10	***
Compaction treatment	-0.02	4.00E-03	-4.44	1.59E-05	***
Node	-0.01	3.00E-03	-1.50	1.35E-01	
Genotype	2.00E-03	3.00E-03	0.49	6.27E-01	
Thickening	-0.05	0.12	-0.40	6.92E-01	

Table S4 – Summary of ANCOVA for the effect of field site, compaction treatment and thickening on rooting depth D_{75}. *** level of significance at $p \leq 0.001$ and * level of significance at $p \leq 0.05$.

	F-value	p-value	
(Intercept)	5288.65	<2.2E-16	***
Field site	84.65	<2.2E-16	***
Compaction treatment	42.76	6.81E-10	***
Thickening	1.30	0.26	
Field site:Compaction Treatment	5.62	0.02	*
Field site:Thickening	5.77	0.02	*
Compaction treatment:Thickening	1.71	0.20	
Field site:Compaction treatment:Thickening	0.76	0.38	

Table S5 – Pearson correlations for anatomical traits and D_{75}. *** level of significance at $p \leq 0.001$, ** level of significance at $p \leq 0.01$ and * level of significance at $p \leq 0.05$. (A) for correlations within node 3 and (B) for correlations within node 4. Abbreviations are as follows; RCSA: Root cross sectional area, TSA: Total stele area, TCA: Total cortical area, TCA/TSA: Ratio cortex to stele, TCA/RCSA: Ratio cortex to cross sectional area, TSA/RCSA: Ratio stele to cross sectional area, CF: cell file number, IN: inner cortical region cell area, MID: middle cortical region cell area, OUT: outer cortical region cell area, AA: aerenchyma area, AA/TCA: ratio of cortex taken up by aerenchyma, AA/RCSA: ratio of cross sectional area taken up by aerenchyma, nonAA: non-aerenchyma cortical area, D_{75}: rooting depth within a 60 cm core above which 75% of the root length of coarse (> 1mm diameter) is found.

A

	RCSA	TSA	TCA	TCA/TSA	TCA/RCSA	TSA/RCSA	CF	IN	MID	OUT	AA	AA/TCA	AA/RCSA	nonAA	D_{75}
RCSA	1.00														
TSA	0.84 **	1.00													
TCA	0.98 **	0.71 **	1.00												
TCA/TSA	-0.05	-0.55 **	0.14	1.00											
TCA/RCSA	-0.09	-0.59 **	0.11	0.97 **	1.00										
TSA/RCSA	0.09	0.59 **	-0.11	-0.97 **	-1.00	1.00									
CF	0.46 **	0.49 **	0.42 **	-0.21 *	-0.21	0.21	1.00								
IN	0.48 **	0.17	0.55 **	0.43 **	0.41 **	-0.41 **	-0.02	1.00							
MID	0.45 **	0.05	0.56 **	0.55 **	0.55 **	-0.55 **	-0.24 *	0.77 **	1.00						
OUT	0.10	-0.29 **	0.24 *	0.67 **	0.65 **	-0.65 **	-0.40 **	0.60 **	0.78 **	1.00					
AA	0.30 **	0.13	0.34 **	0.12	0.13	-0.13	-0.10	0.18	0.43 **	0.27 *	1.00				
AA/TCA	-0.03	-0.10	0.00	0.05	0.08	-0.08	-0.20	0.02	0.25 *	0.19	0.89 **	1.00			
AA/RCSA	-0.03	-0.13	0.01	0.12	0.15	-0.15	-0.22	0.05	0.28 **	0.23 *	0.90***	1.00 **	1.00		
nonAA	0.88 **	0.68 **	0.88 **	0.08	0.04	-0.04	0.49 **	0.49 **	0.36 **	0.11	-0.15	-0.45 **	-0.44 **	1.00	
D_{75}	-0.08	-0.15	-0.05	0.11	0.17	-0.17	-0.40 **	0.01	0.30 *	0.23 *	0.24 *	0.20	0.21 *	-0.17	1.00

B

	RCSA	TSA	TCA	TCA/TSA	TCA/RCSA	TSA/RCSA	CF	IN	MID	OUT	AA	AA/TCA	AA/RCSA	nonAA	D_{75}
RCSA	1.00														
TSA	0.88 **	1.00													
TCA	0.98 **	0.75 **	1.00												
TCA/TSA	-0.20	-0.61 **	0.00	1.00											
TCA/RCSA	-0.19	-0.63 **	0.02	0.97 **	1.00										
TSA/RCSA	0.19	0.63 **	-0.02	-0.97 **	-1.00 **	1.00									
CF	0.55 **	0.57 **	0.49 **	-0.23 *	-0.27	0.27 *	1.00								
IN	0.57 **	0.29 *	0.65 **	0.32 *	0.33 *	-0.33 *	0.10	1.00							
MID	0.43 **	0.08	0.56 **	0.50 **	0.54 **	-0.54 **	-0.18	0.79 **	1.00						
OUT	0.12	-0.21 *	0.26 *	0.66 **	0.65 **	-0.65 **	-0.30 *	0.51 **	0.68 **	1.00					
AA	0.00	-0.12	0.06	0.22 *	0.24 *	-0.24 *	-0.22 *	-0.02	0.31 *	0.14	1.00				
AA/TCA	-0.32 *	-0.34 *	-0.28 *	0.14	0.17	-0.17	-0.41 **	-0.26 *	0.07	0.01	0.88 **	1.00			
AA/RCSA	-0.33 *	-0.38 **	-0.28 *	0.20	0.23 *	-0.23 *	-0.43 **	-0.24 *	0.11	0.06	0.88 **	1.00 **	1.00		
nonAA	0.91 **	0.75 **	0.91 **	-0.08	-0.07	0.07	0.55 **	0.62 **	0.40 **	0.20	-0.35 **	-0.62 **	-0.62 **	1.00	
D_{75}	-0.22 *	-0.28 *	-0.18	0.11	0.15	-0.15	-0.26 *	-0.12	0.14	0.08	0.28 *	0.33 *	0.33 *	-0.26 *	1.00

Table S6 – Summary of stepwise multiple regression models for node 3. *** level of significance at $p \leq 0.001$, ** level of significance at $p \leq 0.01$ and * level of significance at $p \leq 0.05$. Abbreviations are as follows: D_{75}: rooting depth within a 60 cm core above which 75% of the coarse (>1 mm diameter) roots are found, CF: cell file number, MID: middle cortical region cell area, OUT: outer cortical region cell area, AA: aerenchyma area, AA/RCSA: ratio of cross sectional area taken up by aerenchyma.

Multiple linear regression including all preselected traits:
These preselected traits are across tissues (AA, AA/RCSA) and cellular traits (CF, MID, OUT)

Model 1: D_{75} ~ CF + MID + OUT + AA + AA/RCSA

	Estimate	SE	t-value	p-value	
(Intercept)	51.49	13.26	3.88	2.09E-04	***
CF	-3.70	1.00	-3.71	3.75E-04	***
MID	0.01	0.00	1.52	0.13	
OUT	0.00	0.00	-1.12	0.27	
AA	17.38	18.94	0.92	0.36	
AA/RCSA	-18.44	36.10	-0.51	0.61	
Multiple R^2	0.24				
Adjusted R^2	0.19				
p-value	4.85E-03 ***				

Stepwise linear regression of model including all preselected traits:

Model 2: D_{75} ~ CF + MID

	Estimate	SE	t-value	p-value	
(Intercept)	43.96	11.36	3.87	2.14E-04	***
CF	-3.13	0.89	-3.52	6.93E-03	***
MID	4.67E-03	2.22E-03	2.10	0.04	*
Multiple R^2	0.20				
Adjusted R^2	0.19				
p-value	6.65E-05 ***				

Multiple linear regression including preselected tissue traits:

Model 3: D_{75} ~ AA + AA/RCSA

	Estimate	SE	t-value	p-value	
(Intercept)	13.92	2.33	5.98	5.30E-08	***
AA	15.90	17.24	0.92	0.36	
AA/RCSA	2.28	34.76	0.07	0.95	
Multiple R^2	0.06				
Adjusted R^2	0.03				
p-value	9.16E-02				

Stepwise linear regression of model including preselected tissue traits:

Model 4: D_{75} ~ AA

	Estimate	SE	t-value	p-value	
(Intercept)	13.98	2.09	6.70	2.17E-09	***
AA	16.92	7.59	2.23	2.84E-02	*
Multiple R^2	0.06				
Adjusted R^2	0.04				
p-value	2.84E-02 *				

Multiple linear regression including preselected cellular traits:

Model 5: D_{75} ~ CF + MID + OUT

	Estimate	SE	t-value	p-value	
(Intercept)	48.79	11.85	4.12	8.97E-05	***
CF	-3.58	0.94	-3.79	2.82E-04	***
MID	0.01	3.43E-03	2.40	1.86E-02	*
OUT	-0.01	4.35E-03	-1.36	0.18	
Multiple R^2	0.22				
Adjusted R^2	0.19				
p-value	1.08E-04 ***				

Stepwise linear regression of model including preselected cellular traits:

Model 6: D_{75} ~ CF + MID

See model 2 for summary

Table S7 – Summary of stepwise multiple regression models for node 4. *** level of significance at p≤0.001, ** level of significance at p ≤ 0.01 and * level of significance at p ≤ 0.05. Abbreviations are as follows: D_{75}: rooting depth within a 60 cm core above which 75% of the coarse (>1 mm diameter) roots are found, RCSA: root cross sectional area, TSA: total stele area, CF: cell file number, AA: aerenchyma area, AA/TCA: Ratio of cortex taken up by aerenchyma, AA/RCSA: ratio of cross sectional area taken up by aerenchyma, nonAA: non-aerenchyma cortical area.

Multiple linear regression including all preselected traits:
These preselected traits are across tissues (RCSA, TSA, AA, AA/TCA and AA/RCSA) and just one cellular trait (CF)

Model 1: D_{75} ~ RCSA + TSA + CF + AA + AA/TCA + AA/RCSA + nonAA

	Estimate	SE	t-value	p-value	
(Intercept)	33.46	15.10	2.22	2.96E-02	*
RCSA	-4.73	32.82	-0.14	0.89	
TSA	-15.35	42.22	-0.36	0.72	
CF	-1.06	1.12	-0.95	0.35	
AA	17.25	30.31	0.57	0.57	
AA/TCA	250.59	232.89	1.08	0.29	
AA/RCSA	-326.05	325.73	-1.00	0.32	
nonAA	9.18	30.13	0.31	0.76	
Multiple R^2	0.18				
Adjusted R^2	0.10				
p-value	2.73E-02 *				

Stepwise linear regression of model including all preselected traits:

Model 2: D_{75} ~ TSA + AA/RCSA

	Estimate	SE	t-value	p-value	
(Intercept)	20.11	4.95	4.06	1.09E-04	***
TSA	-9.00	5.18	-1.74	8.61E-02	
AA/TCA	27.46	11.12	2.47	1.56E-02	*
Multiple R^2	0.14				
Adjusted R^2	0.12				
p-value	1.83E-03 **				

Multiple linear regression including preselected tissue traits:

Model 3: D_{75} ~ RCSA + TSA + AA + AA/TCA + AA/RCSA + nonAA

	Estimate	SE	t-value	p-value	
(Intercept)	21.05	7.51	2.80	6.32E-03	**
RCSA	-8.82	32.52	-0.27	0.79	
TSA	-11.41	41.98	-0.27	0.79	
AA	18.23	30.27	0.60	0.55	
AA/TCA	207.08	228.17	0.91	0.37	
AA/RCSA	-256.91	317.24	-0.81	0.42	
nonAA	12.46	29.91	0.42	0.68	
Multiple R^2	0.17				
Adjusted R^2	0.10				
p-value	2.07E-02 *				

Stepwise linear regression of model including preselected tissue traits:

Model 4: D_{75} ~ TSA + AA/RCSA

See model 2 for summary

Multiple linear regression including preselected cellular traits:

Model 5: D_{75} ~ CF

	Estimate	SE	t-value	p-value	
(Intercept)	46.00	11.36	4.05	1.13E-04	***
CF	-2.18	0.87	-2.50	1.44E-02	*
Multiple R^2	0.07				
Adjusted R^2	0.06				
p-value	1.44E-02 *				

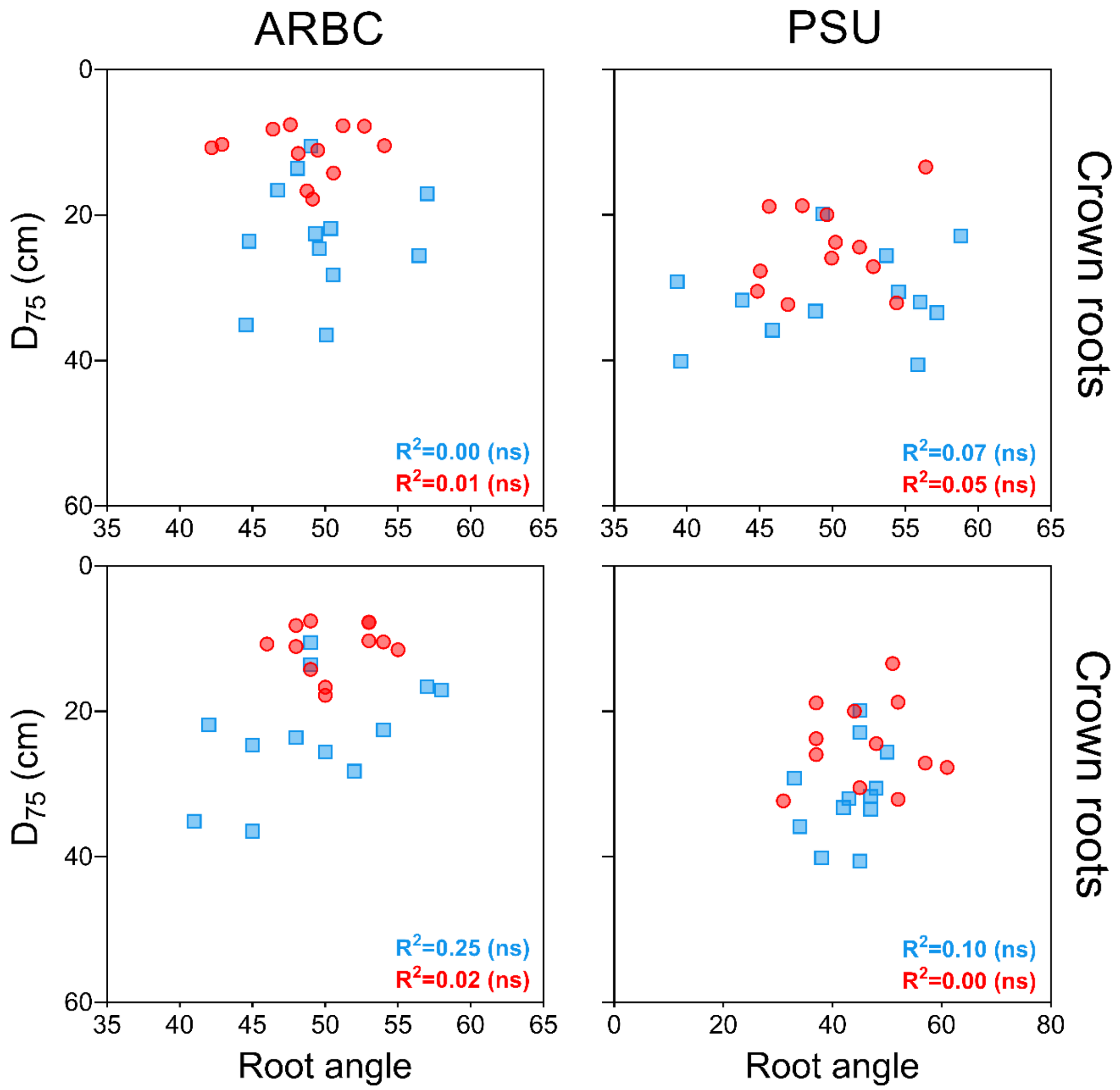

Fig S1 – Relationship between root angle and D_{75}. No relationship was found for crown root angle or brace root angle with D_{75} under both compacted (red) and non-compacted (blue) conditions. Ns stands for non-significant.

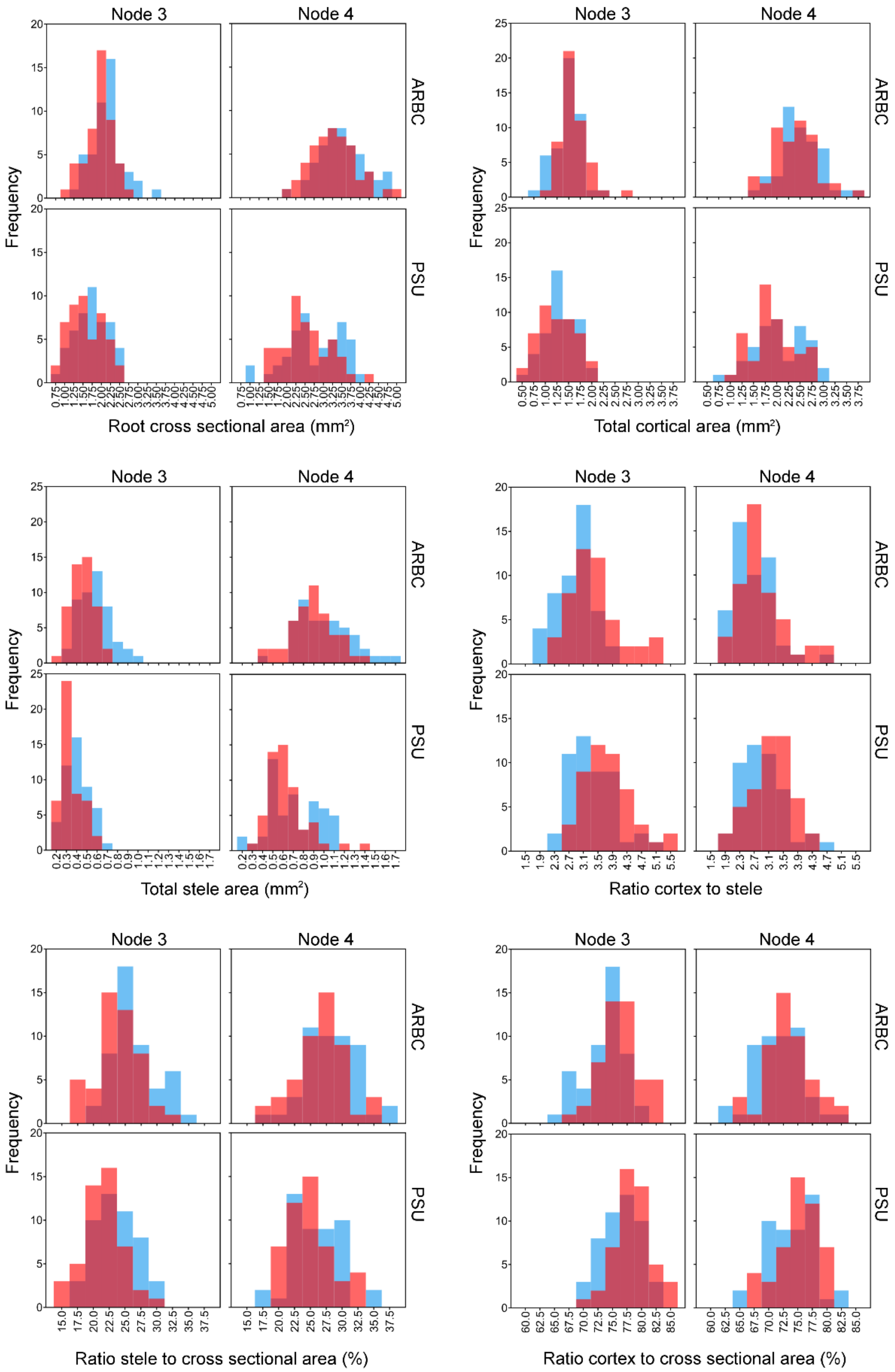

Figure S2 – Histograms for each anatomical trait measured within for each field site and node. Compacted data in red, non-compacted data in blue. ARBC stands for the Apache Root Biology field site and PSU stands for the Russel E. Larson Agricultural Research Center at Pennsyllvania State University. (page 1/3)

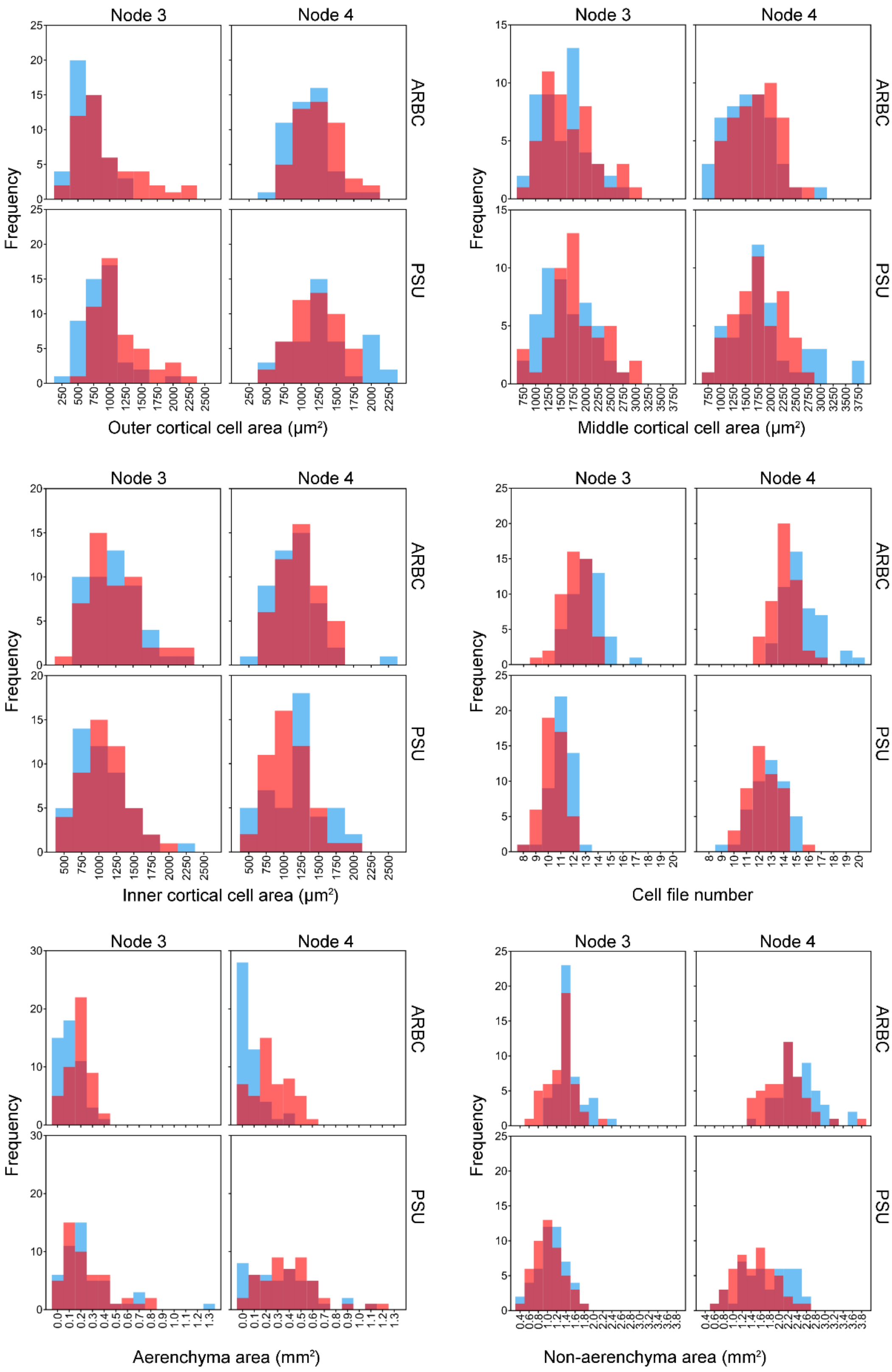

Figure S2 – Histograms for each anatomical trait measured within each field season and divided by node. Compacted data in red, non-compacted data in blue. ARBC stands for the Apache Root Biology field site and PSU stands for the Russel E. Larson Agricultural Research Center at Pennsyllvania State University. (page 2/3)

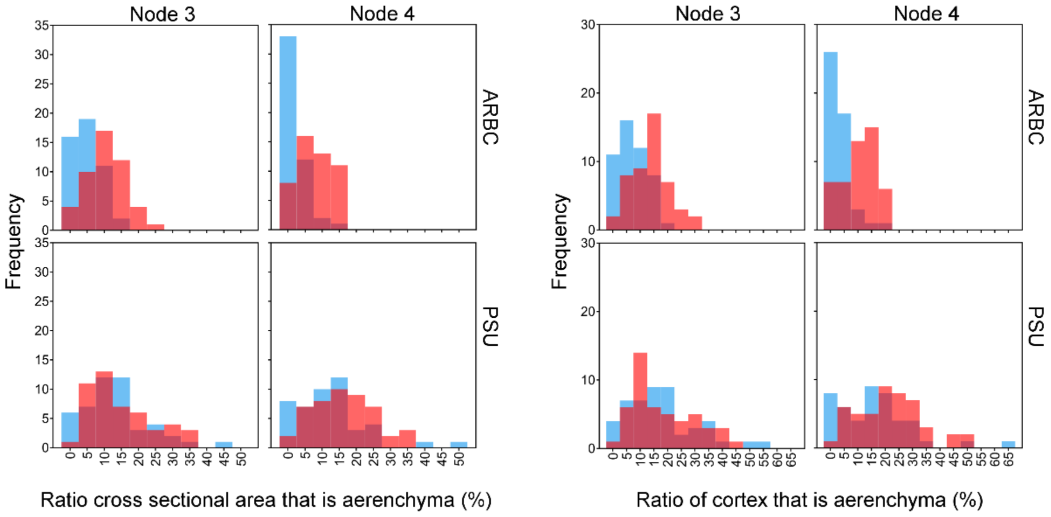

Figure S2 – Histograms for each anatomical trait measured within each field season and divided by node. Compacted data in red, non-compacted data in blue. ARBC stands for the Apache Root Biology field site and PSU stands for the Russel E. Larson Agricultural Research Center at Pennsyllvania State University. (page 3/3)

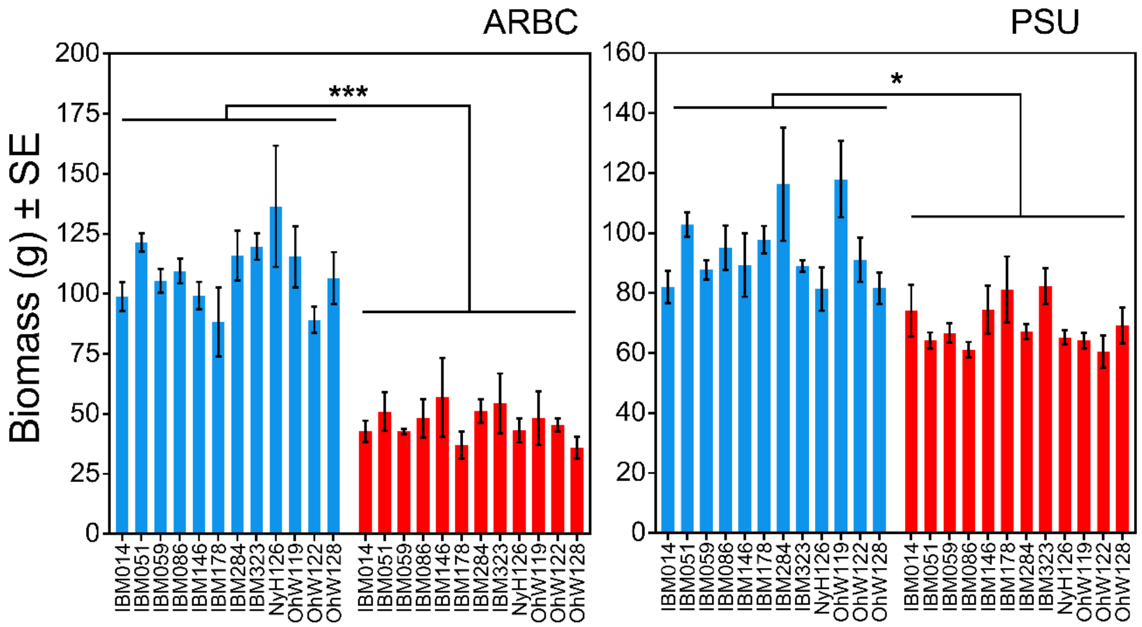

Figure S3 – Biomass ± SE at both field sites under compacted (red) and non-compacted (blue) conditions for each field site. Significant reduction in biomass was found between treatments (significance levels: *** p ≤ 0.001 and * p ≤ 0.05) No differences were detected between genotypes within the compaction or non-compaction plots.

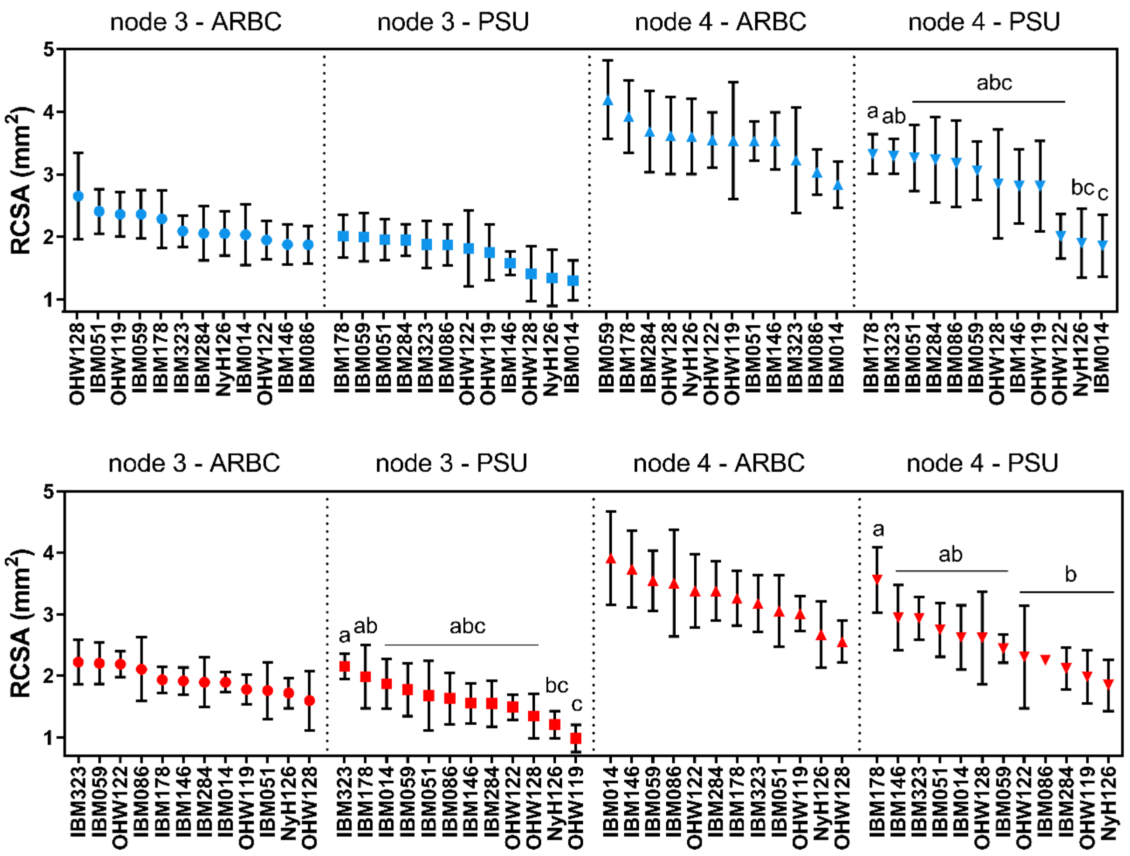

Figure S4 – Differences between genotypes for the trait root cross sectional area (RCSA) within each node and genotype combination.

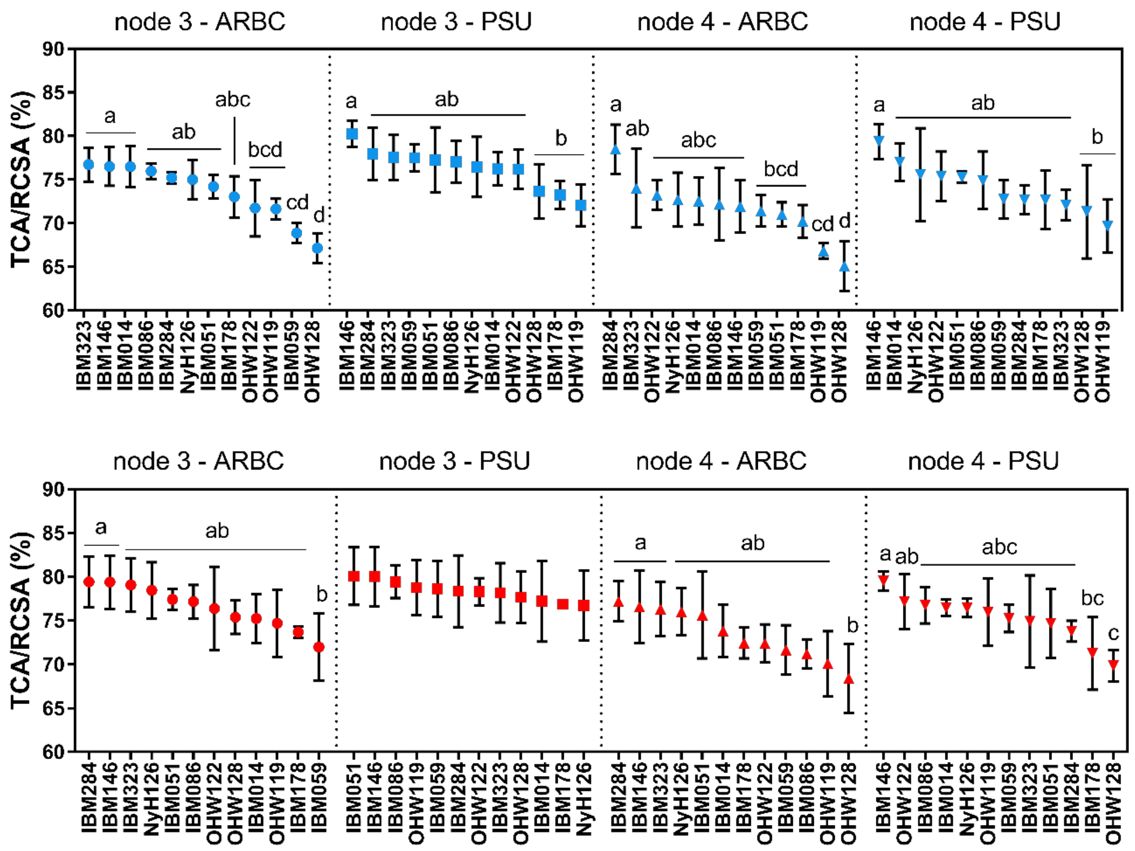

Figure S5 – Differences between genotypes for the trait root ratio of total cortical area to cross sectional area (TCA/RCSA) within each node and genotype combination.

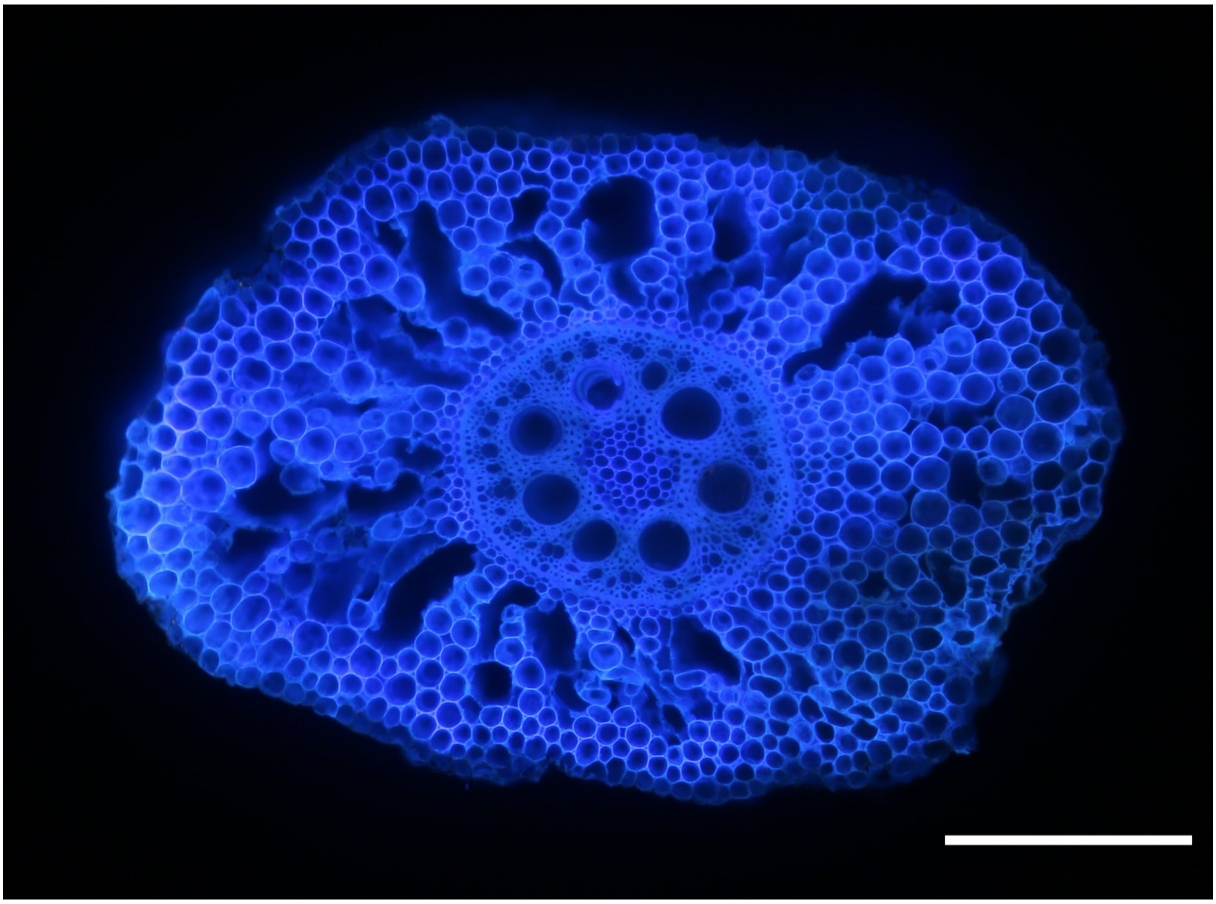

Figure S6 – Example of irregularly shaped root section of a root grown under compacted conditions. Root taken from node 3, scale bar at 500 μm.
